# Participant-Independent Classification of Autism-Related Visual Attention Patterns from Eye-Tracking Scanpath Images Using a Global–Local Fusion Network

**DOI:** 10.3390/jemr19040085

**Published:** 2026-08-10

**Authors:** Kun Zhang, Junling Kong, Junhui Zhang, Shuo Zhang, Jingying Chen

**Affiliations:** 1 National Engineering Research Center for E-Learning, Faculty of Artificial Intelligence in Education, Central China Normal University, Wuhan 430079, China; zhk@mail.ccnu.edu.cn (K.Z.); 2024114349@mails.ccnu.edu.cn (J.K.); 2025124770@mails.ccnu.edu.cn (J.Z.); zhangshuo111@mails.ccnu.edu.cn (S.Z.); 2 National Engineering Research Center of Educational Big Data, Faculty of Artificial Intelligence in Education, Central China Normal University, Wuhan 430079, China

**Keywords:** autism spectrum disorder, eye-tracking scanpath, visual attention, global–local collaborative fusion network, cross-subject generalization

## Abstract

Children with autism spectrum disorder (ASD) often exhibit atypical patterns of visual attention allocation and social-cue processing. Eye-tracking scanpath (ETSP) retains information about fixation points, saccade paths and their temporal changes in the form of images, providing an intuitive and computable data representation for analyzing ASD-related visual attention patterns. However, in ASD auxiliary identification studies, the same participant often generates multiple eye-tracking recordings or multiple visual representation samples. If participant independence is not properly considered during model evaluation, the training and test sets may share individualized eye-movement patterns from the same child. In such cases, the model may learn subject-specific characteristics rather than stable and transferable ASD-related visual attention features, leading to an overestimation of its recognition ability on unseen participants. To address this issue, we propose a Global–Local Collaborative Fusion Network (GLCF-Net) under a strict participant-independent splitting protocol. Specifically, the proposed method first maps ETSP images into patch token sequences through a shared Patch Embedding layer. A CNN-based local branch is then used to extract local trajectory morphology, path density, and spatial neighborhood structure, while a ViT-based global branch models cross-region gaze transitions and the overall attention distribution. Finally, a gated adaptive fusion module dynamically integrates local and global information to enhance the representation of stable visual attention features. In the primary repeated stratified five-fold participant-level evaluation, averaging the two out-of-fold probabilities for each participant yielded an Accuracy of 87.0% and a ROC-AUC of 93.7%; the original participant split, retained as a secondary analysis, yielded an Accuracy of 83.52% and a ROC-AUC of 90.27%. Under the reported frozen-backbone configurations, the model also showed a balanced pattern across Accuracy, Recall, and F1-score. These results characterize performance for unseen participants within the same dataset and acquisition conditions.

## 1. Introduction

Autism spectrum disorder (ASD) is a neurodevelopmental disorder characterized primarily by social communication impairments, restricted interests, and repetitive stereotyped behaviors, with substantial individual variability and complex etiological mechanisms [1]. Epidemiological studies have shown that the global prevalence and disease burden of ASD have generally increased in recent years, with a particularly pronounced burden among young children, highlighting the growing need for early screening and intervention [2]. Traditional ASD screening methods mainly rely on caregiver-completed questionnaires or clinical observation, and they remain limited by subjectivity, delayed identification, and time-consuming procedures [1,3]. Therefore, developing objective, timely, and efficient auxiliary screening methods for ASD is of great significance for early identification and improved prognosis.

In recent years, with rapid advances in eye-tracking technology, computer vision, and artificial intelligence (AI), ASD auxiliary screening based on objective behavioral signals has become an increasingly active research topic. Eye-movement behavior can record learners’ fixation locations, region transitions, and temporal changes in visual scenes [4]. However, traditional eye-tracking analysis methods mostly depend on statistical features such as fixation duration, fixation count, first fixation time, and dwell proportion within areas of interest, often combined with conventional machine learning algorithms for ASD auxiliary classification [3,4,5,6,7]. These methods still require considerable manual processing and are limited in their ability to preserve the spatial structure and dynamic evolution of eye-movement behavior. Eye-tracking scanpaths (ETSP), which transform fixation points and saccadic paths into visual images, contain fine-grained information such as local trajectory morphology and texture distribution, while also encoding global semantic information such as overall fixation patterns, spatial layout, and cross-region dependencies. ETSPs have therefore become an important representation for visual attention modeling.

Previous studies have demonstrated that ETSP scanpath images can effectively support ASD identification and classification. Carette et al. [8] transformed scanpaths into visual representations and verified the feasibility of using scanpath images for modeling visual attention patterns. The same research team later released an eye-tracking dataset for ASD research, providing a reproducible data basis for subsequent studies [9]. Building on this dataset, Cilia et al. [10] further validated the classification performance of CNNs, indicating the importance of local spatial texture features for visual attention pattern analysis. Kanhirakadavath et al. [11] improved the representation of ASD-related visual attention differences through image augmentation and DNN-based modeling. Subsequently, Alsaidi et al. [12] proposed a T-CNN architecture to enhance spatial feature extraction.

Benabderrahmane et al. [13] combined scanpath images with sequential eye-movement signals and used GRU networks to model temporal dependencies. Mousli et al. [14] used supervised contrastive learning to pretrain an encoder and then fine-tuned the classifier, improving feature stability under limited training samples and, to some extent, classification performance in cross-individual scenarios. Al-Adhaileh et al. [15] further combined CNN and LSTM models for joint spatial–temporal modeling and achieved high classification performance on public datasets.

The broader eye-movement literature provides the behavioral context for these computational studies. Attention to socially informative regions, including the eye area, can follow different developmental trajectories in children later diagnosed with ASD [16]. Meta-analytic evidence indicates that gaze differences between ASD and non-ASD groups are generally heterogeneous and depend on whether the displayed information is social or nonsocial [17]. Stimulus type also materially affects measured social attention [18], while scanpath-level analyses have reported both reduced social preference and increased persistence toward nonsocial content [19]. Accordingly, the local branch of GLCF-Net may capture short-range trajectory density and morphology, whereas the global branch may capture broader spatial allocation and cross-region transitions; these computational features should not be interpreted as direct biological or diagnostic markers.

However, ASD is not a homogeneous condition; it involves substantial phenotypic heterogeneity and varied clinical outcomes. Eye-gaze profiles across children can be discrete and highly heterogeneous [20,21]. AI models that rely only on shallow features often fail to cope with cross-subject behavioral variability induced by such heterogeneity, resulting in a marked decline in generalization performance when tested on unseen participants [22]. In the ETSP dataset, each child is typically associated with multiple scanpath images, which may share relatively stable individualized gaze habits, saccadic speed, trajectory density, and spatial preferences. If different images from the same participant appear simultaneously in the training and test sets, the model may learn participant-specific eye-movement patterns rather than transferable ASD-related visual attention patterns. Consequently, when facing unseen participants, the model may suffer from poor generalization, even though its reported performance appears falsely high.

For example, Cilia et al. [10] further adopted participant-ID-based independent splitting, ensuring that ETSP images from the same participant were strictly assigned to the same subset. Their results showed that model accuracy decreased from 90% to 71%. In addition, the original augmentation strategy used by Kanhirakadavath et al. [11] involved sample-level data leakage, where augmented images appeared simultaneously in the training and validation sets, leading to overly optimistic performance estimates. When the same study further evaluated a mini dataset containing one trial from each of 59 participants, the DNN model achieved an overall Accuracy of 72.88%. Moreover, although some subsequent studies did not use data augmentation and thereby avoided augmentation-induced leakage, they still did not sufficiently emphasize the principle of participant-independent splitting. Therefore, studies reporting accuracies above 90% in the existing literature may have limited practical significance if their generalization ability is weak [12,13,14,15].

In summary, insufficient model generalization is a common issue in ASD identification research. In ETSP scanpath datasets and related studies, two types of problems may lead to inflated evaluation results. The first is sample-level leakage: researchers augment the original images before performing random cross-validation on the entire augmented dataset, causing augmented copies of the same original image to be assigned to different training and validation folds. The second is the cross-subject generalization problem: even without augmentation, if the training and test sets are not divided by participant, different original images from the same participant may randomly appear in both sets. In this situation, the model has already “seen” the participant’s eye-movement pattern during training, thereby overestimating cross-individual generalization ability.

Eye-tracking scanpath images are essentially visual representations generated by mapping fixation points, saccadic paths, and their temporal changes. Local trajectory morphology, line density, color variation, and spatial neighborhood relationships between adjacent patches can reflect fine-grained differences in short-term visual exploration. In contrast, overall fixation layout, cross-region transition patterns, and spatial dependencies among different areas of interest reflect higher-level visual attention strategies.

In computer vision, CNNs are well suited for extracting local details, whereas Vision Transformers (ViTs) can complement them by capturing global context [23,24,25,26]. The complementarity between CNNs and ViTs has been widely discussed. For example, in multicellular morphology classification related to Alzheimer’s disease, researchers fused EfficientNet and ViT modules and demonstrated the effectiveness of collaborative modeling of local texture and long-range context for improving generalization on limited datasets [27]. Li et al. [24] proposed a parallel-branch CNN-Transformer module for change detection, using a convolutional branch to extract local features and a Transformer branch to extract global features. Through dependent and concurrent fusion, they constructed multiscale global–local representations.

Based on the above analysis, and considering that ETSP images contain both local saccadic trajectory details and global fixation distribution patterns, we extend the multiscale collaborative idea of CNN–ViT modeling to the ETSP auxiliary identification task and propose the Global–Local Collaborative Fusion Network, namely GLCF-Net. By combining local detail modeling with global relationship modeling, GLCF-Net can more comprehensively extract stable discriminative features from ETSP images, thereby improving within-dataset cross-participant generalization under participant-independent splitting.

The primary hypothesis was that global–local fusion would improve Accuracy and ROC-AUC relative to the matched ViT-B/16 configuration under participant-independent evaluation. Repeated grouped participant folds were used to characterize sensitivity to participant partitioning, while ablations assessed the contributions of the gate and CLS readout under participant-level aggregation. Comparisons with the CNN baselines were treated as reference comparisons because their trainable capacities differed. Throughout this article, “generalization” refers to unseen participants from the same dataset and acquisition conditions; independent cohorts, centers, eye trackers, stimuli, age groups, and demographic populations were not evaluated.

## 2. Materials and Methods

### 2.1. Dataset

**Figure 1:**
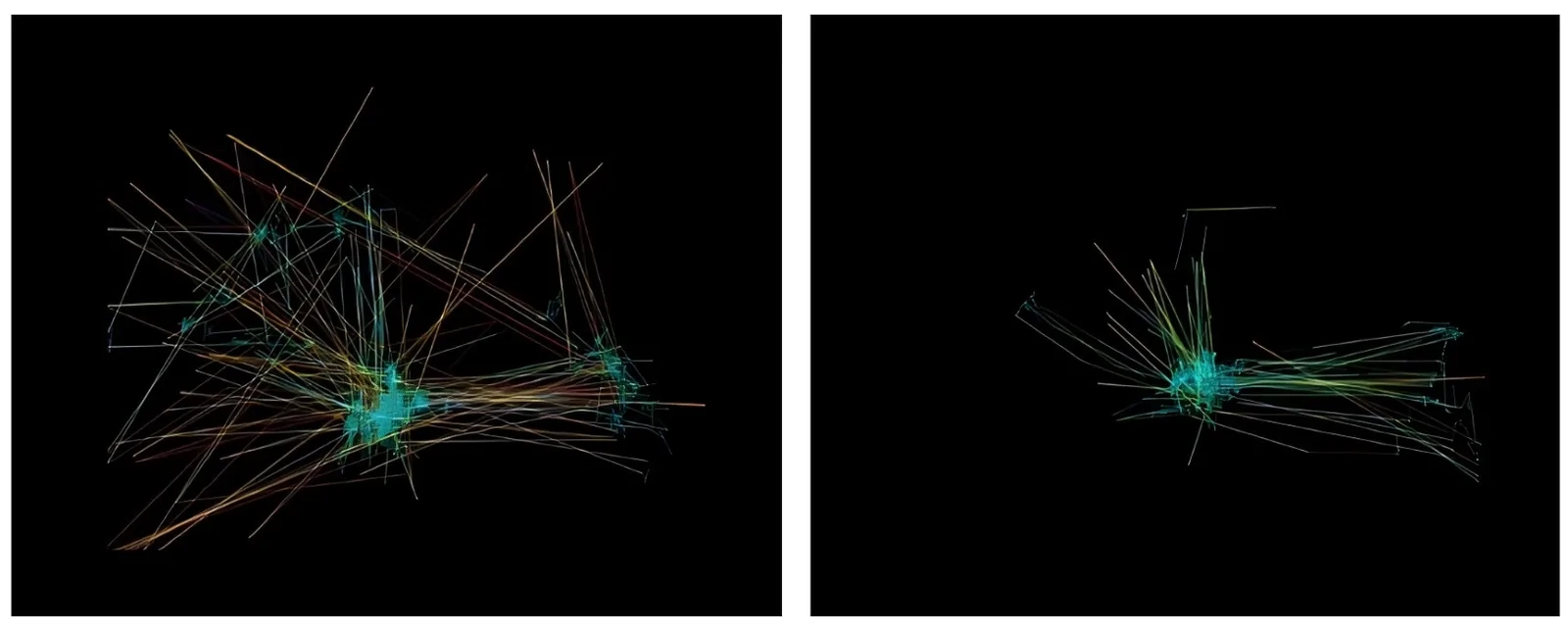
Visualization of eye-tracking scan paths. The (**left**) image shows a participant diagnosed with autism spectrum disorder (ASD), while the (**right**) image shows a typically developing (TD) participant.RGB channels map velocity, acceleration, and jerk over time, respectively. Velocity varies from black (low) to red (high); acceleration and jerk are encoded via green and blue gradients.

We used a public ETSP scanpath dataset as the research dataset. This dataset has been used in several ASD eye-tracking identification studies and provides good reproducibility and comparability. The dataset was selected for two main reasons. First, the participants were school-age children, which is consistent with the objective of this study to analyze visual attention patterns in children with ASD. Second, the dataset contains RGB scanpath images generated from eye-movement trajectories. By encoding dynamic information such as velocity and acceleration into color channels, these images preserve the spatial distribution of eye movements while incorporating certain temporal changes. Figure 1 presents representative scanpaths from an ASD participant (left) and a TD participant (right). The two examples differ in trajectory morphology, but this illustration only characterizes the data format and does not establish group-level differences.

The public ETSP dataset used in this study is available from the Figshare data repository: https://figshare.com/s/5d4f93395cc49d01e2bd (accessed on 26 March 2026). It includes 59 school-age children, consisting of 38 male and 21 female participants, with an average age of approximately 7.88 years. Among them, 29 were children with ASD and 30 were typically developing (TD) children. Through eye-tracking visualization, a total of 547 images were generated, including 219 ASD images and 328 TD images, with a resolution of 640 × 480 pixels. The images are stored in two separate subfolders, one for ASD participants and the other for TD participants. In addition, the metadata include CSV and JSON files that record participant information and map each image to a unique participant ID, which is used to identify each child participant in the experiment. Of the 59 metadata records, 54 participants (26 ASD and 28 TD) had at least one released ETSP image. The original fixed-split experiment used a class-balanced subset of 52 participants (26 ASD and 26 TD); the two additional image-contributing TD participants, TD-47 and TD-57, were not included in that original subset. The repeated participant-level evaluation included all 54 image-contributing participants, with each participant’s images treated as repeated observations. The remaining five metadata records had no corresponding released image and were therefore not included in image-based evaluation.

### 2.2. Data Preprocessing

#### 2.2.1. Data Partition

**Figure 2:**
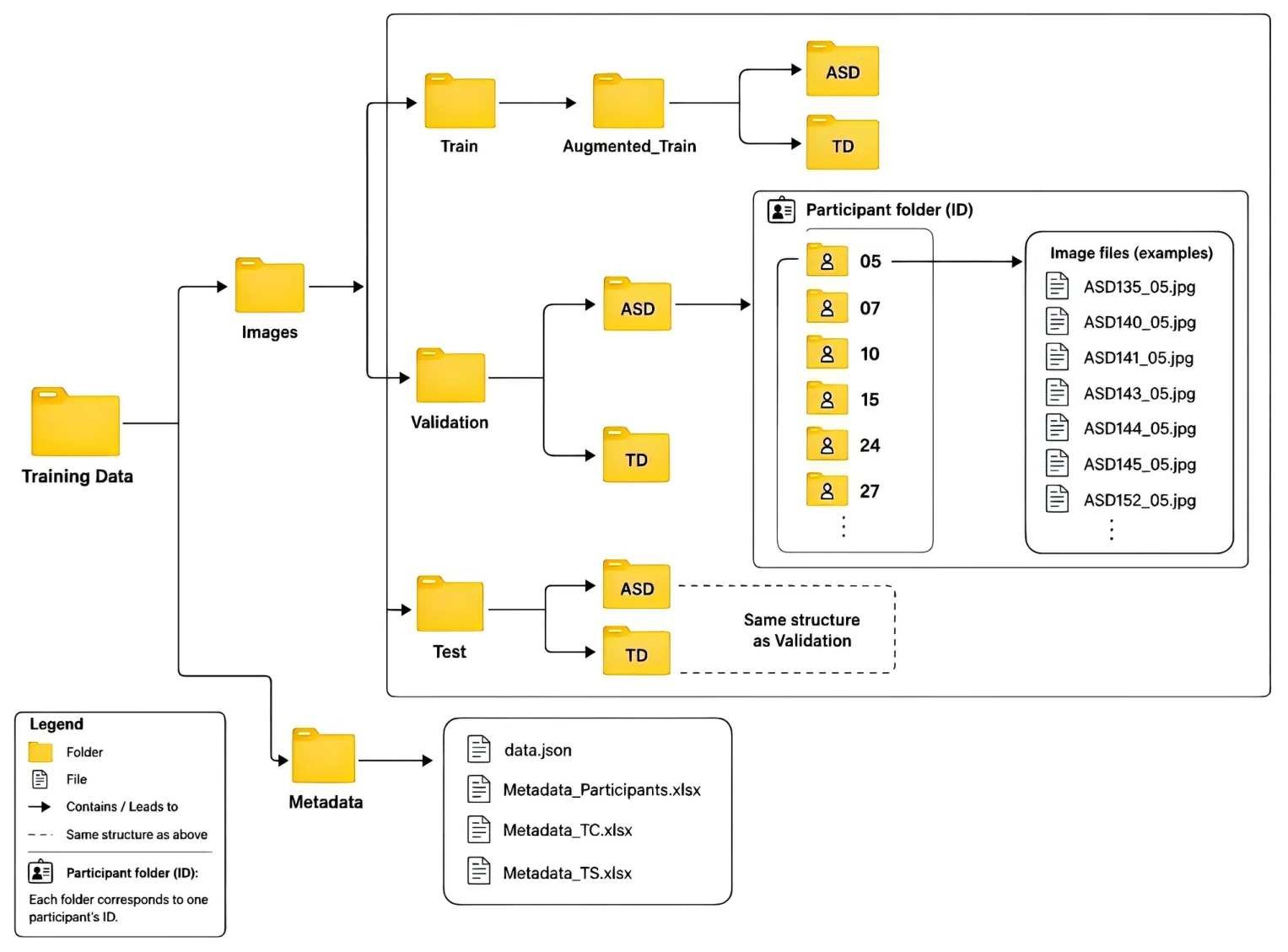
File organization and participant-independent data splitting procedure. All images from the same participant were assigned to only one subset. Data augmentation was applied only to the training set.

To avoid sample-level data leakage, a strict participant-independent splitting strategy was adopted. Figure 2 summarizes the file organization used for participant-independent splitting. The data splitting procedure consisted of the following steps. First, all images were read from the original class directories. Second, the metadata files were used to establish the correspondence between each image and its participant ID. Third, the images were regrouped according to participant ID. Next, the 52 participants in the original fixed-split subset were randomly divided into the training, validation, and test sets at an approximate ratio of 7:1:2 using random seed 42, resulting in 37 training participants (18 ASD and 19 TD), 5 validation participants (3 ASD and 2 TD), and 10 test participants (5 ASD and 5 TD), while ensuring that all original data from the same participant, including ETSP images under all stimuli, appeared in only one subset. Finally, data augmentation was performed only on the training set, while the validation and test sets retained the original images and had no image-level overlap with the training set throughout the entire process.

#### 2.2.2. Data Augmentation

To reduce overfitting under small-sample conditions, data augmentation was applied only to the training set, while the validation and test sets retained the original images. The augmented samples did not cross different data subsets, and neither the same participant nor the augmented versions of the same original image appeared simultaneously in the training set and the validation/test sets.

Considering that the RGB channels of ETSP images encode dynamic eye-movement information rather than natural color appearance, color-space perturbations such as ColorJitter and sample-mixing strategies such as MixUp or CutMix were not adopted. Instead, rotation-based augmentation was used to introduce moderate geometric variations while preserving the overall scanpath topology and trajectory structure. During the original single-split training, each training image was rotated with probability 0.5 using an angle sampled uniformly from −10° to +10°, whereas validation and test images were not rotated. This transformation was applied only to the scanpath images; the stimulus layouts retained their original orientation. Rotation was omitted from the repeated primary comparison and retained in a sensitivity analysis that reproduced the original training configuration.

### 2.3. Model Architecture

#### 2.3.1. Overall Design of GLCF-Net

**Figure 3:**
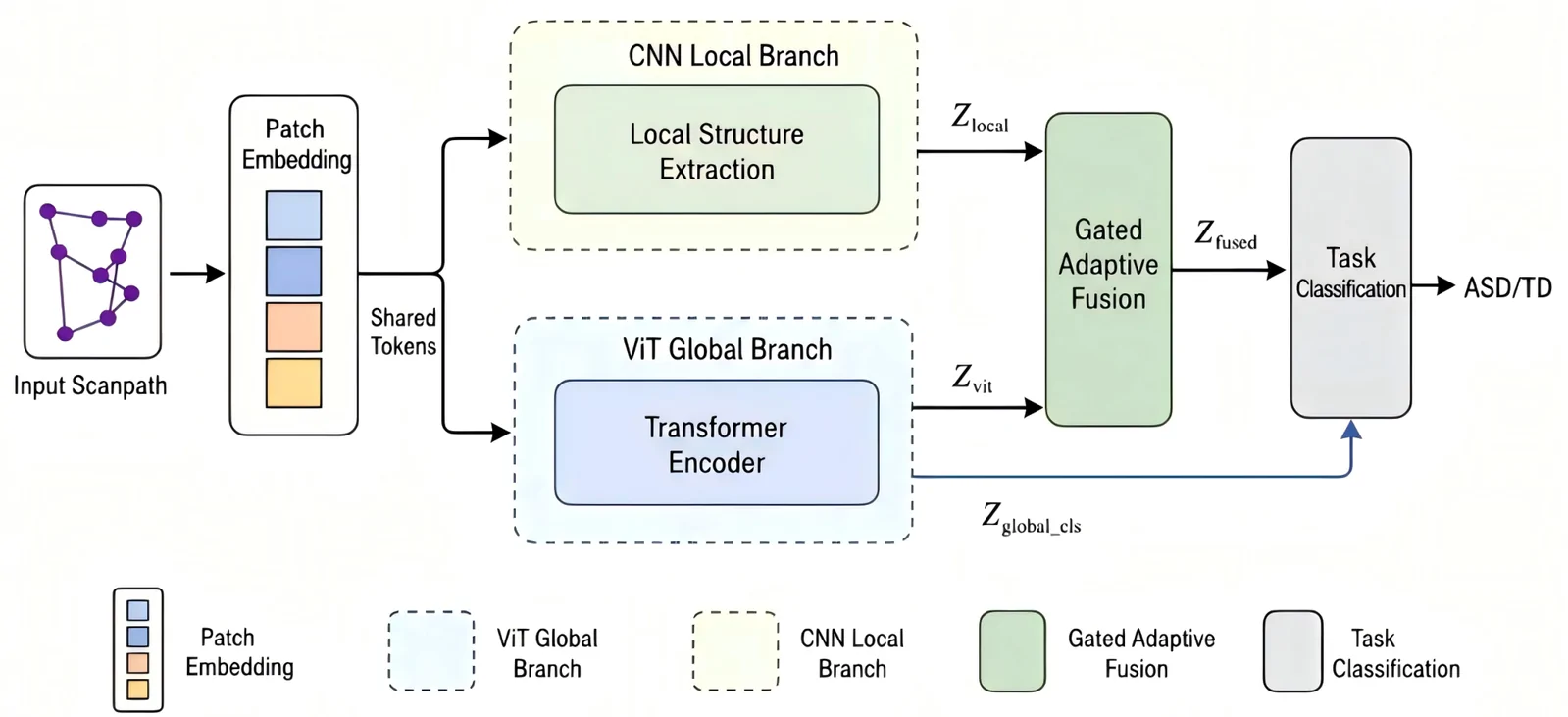
Overall architecture of global–local collaborative fusion network (GLCF-Net).

GLCF-Net first divides the input RGB scanpath image into fixed-size patches and maps them into a patch token sequence through a shared Patch Embedding layer. This sequence serves as the shared input to two branches. On the one hand, the CNN local branch directly reshapes the one-dimensional token sequence into a two-dimensional feature map and extracts local spatial neighborhood structural features through residual convolutional blocks, producing the local feature representation $Z_{\mathrm{local}}$. On the other hand, the ViT global branch prepends a classification token to the patch token sequence, adds positional embeddings, and then models long-range dependencies among different patches through Transformer encoders, producing global contextual patch features $Z_{\mathrm{vit}}$ as well as a classification-token feature $Z_{\mathrm{global}\_\mathrm{cls}}$. In the feature fusion stage, the local and global features are fed into the gated adaptive fusion module to generate fused patch-level features $Z_{\mathrm{fused}}$. The classification module then combines the fused features with the global classification-token feature output by the ViT branch to distinguish ASD and TD samples. The overall architecture of the proposed GLCF-Net is illustrated in Figure 3.

#### 2.3.2. Shared Patch Embedding Layer

**Figure 4:**
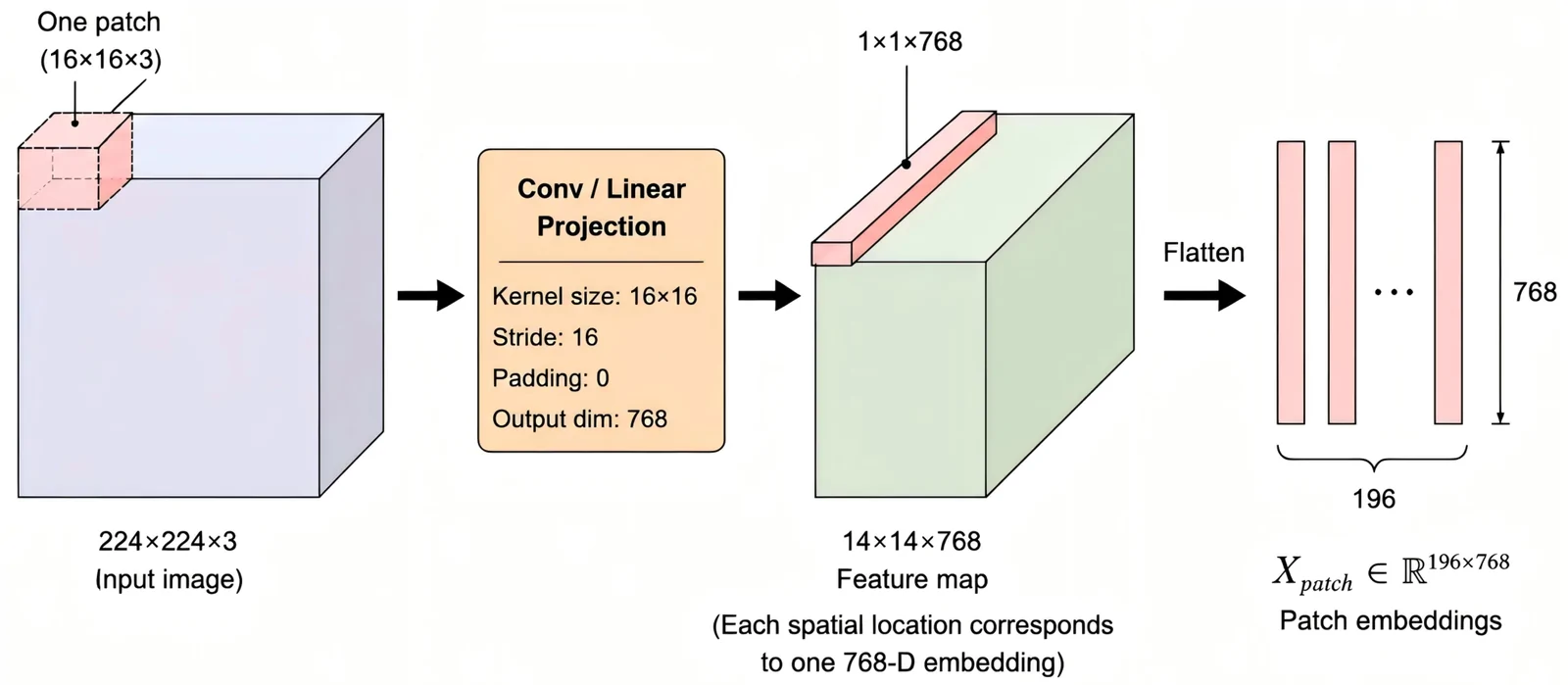
Illustration of Patch Embedding.

To provide a unified input representation for the CNN local branch and the ViT global branch, a shared Patch Embedding layer is introduced before the two branches. This module first divides the input scanpath image into fixed-size patches and maps each patch into the same token representation space, thereby preserving patch-level correspondence between local and global features during subsequent fusion. The detailed operation of this module is illustrated in Figure 4.

At the model input stage, each eye-tracking scanpath image was uniformly resized to 224×224. The input image is denoted as *X*:

(1) $$X\in R^{B\times 3\times H\times W}$$

where *B* denotes the batch size, 3 corresponds to the RGB color channels, and *H* and *W* represent the image height and width, respectively. In this experiment, H=W=224.

Given a patch size of P×P, the input image is divided into *N* non-overlapping patches:

(2) $$N=\frac{HW}{P^{2}}$$

Since the ViT branch adopts ViT-Base/16 as its backbone, the patch size is set to P=16. Therefore, each 224×224 image is divided into 14×14=196 patches.

The Patch Embedding layer then maps each patch into a *d*-dimensional token vector. With the embedding dimension set to d=768, the resulting patch token sequence is obtained as:

(3) $$X_{\mathrm{patch}}=\mathrm{PatchEmbed}\left(X\right)\in R^{B\times N\times d},\;X_{\mathrm{patch}}\in R^{B\times 196\times 768}$$

The shared token sequence is subsequently fed into the CNN local branch and the ViT global branch. The former supplements local spatial structural information within patch neighborhoods, whereas the latter models long-range dependencies among different patches.

#### 2.3.3. CNN Local Branch

**Figure 5:**
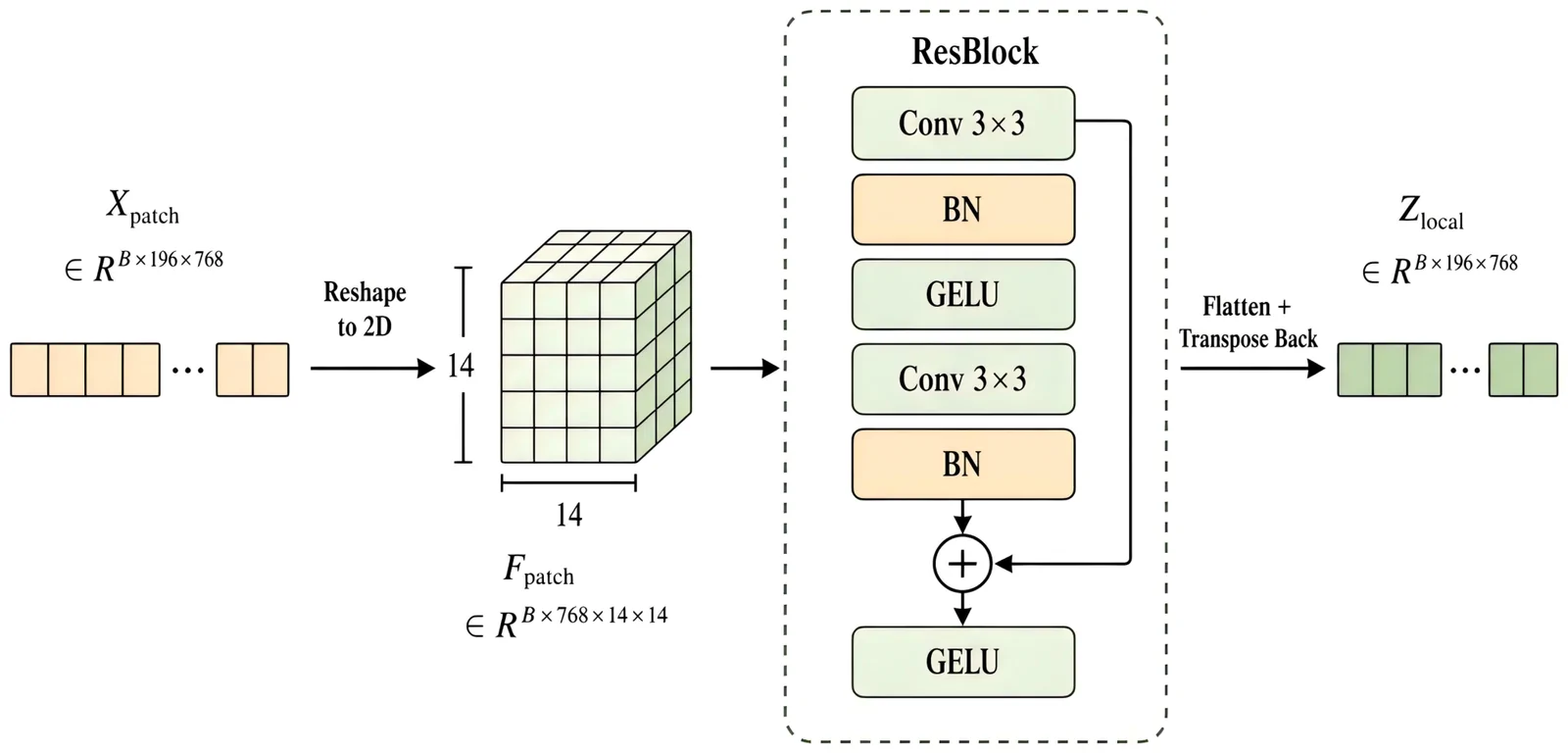
Structure of the convolutional neural network (CNN) local branch.

Figure 5 illustrates how the CNN local branch supplements the shared patch representation with local spatial structure.

The output of the shared Patch Embedding layer, $X_{\mathrm{patch}}$, remains a one-dimensional token sequence. Although each token corresponds to a fixed patch in the input image, the original two-dimensional spatial adjacency among patches is not explicitly exploited in this representation. Therefore, before entering the convolutional module, the token sequence is reshaped into a two-dimensional feature map:

(4) $$F_{\mathrm{patch}}=\mathrm{Reshape}\left(X_{\mathrm{patch}}\right)\in R^{B\times d\times \sqrt{N}\times \sqrt{N}}=R^{B\times 768\times 14\times 14}$$

A lightweight residual convolutional block is then applied to $F_{\mathrm{patch}}$ for local feature enhancement. The residual block consists of two 3×3 convolutional layers, both with 768 input and output channels, a stride of 1, and a padding size of 1, thereby preserving the spatial resolution of the feature map. Each convolutional layer is followed by batch normalization. A GELU activation function is introduced after the first convolutional layer, whereas the output of the second convolutional layer is added to the input feature through a residual connection and then passed through another GELU activation function to obtain the enhanced local representation. This design introduces local receptive fields and neighborhood modeling capability without changing the feature dimensionality, thereby strengthening the representation of local geometric structures that may not be sufficiently captured by the ViT branch. The computation is formulated as:

(5) $$\begin{array}{cc} F_{\mathrm{local}} & =\mathrm{GELU}\left(F_{\mathrm{patch}}+F_{\mathrm{conv}}\left(F_{\mathrm{patch}}\right)\right),\;F_{\mathrm{local}}R^{B\times 768\times 14\times 14},\\ F_{\mathrm{conv}}\left(F_{\mathrm{patch}}\right) & ={\mathrm{BN}}_{2}\left({\mathrm{Conv}}_{3\times 3}^{\left(2\right)}\left(\mathrm{GELU}\left({\mathrm{BN}}_{1}\left({\mathrm{Conv}}_{3\times 3}^{\left(1\right)}\left(F_{\mathrm{patch}}\right)\right)\right)\right)\right). \end{array}$$

Here, ${\mathrm{Conv}}_{3\times 3}^{\left(1\right)}$ and ${\mathrm{Conv}}_{3\times 3}^{\left(2\right)}$ denote the two 3×3 convolutional operations in the residual block, both of which maintain 768 input and output channels. ${\mathrm{BN}}_{1}$ and ${\mathrm{BN}}_{2}$ denote the corresponding batch normalization operations.

To enable subsequent gated fusion with the patch-level features produced by the ViT branch, the two-dimensional feature map is flattened and transposed back into a token sequence:

(6) $$Z_{\mathrm{local}}=\mathrm{Transpose}\left(\mathrm{Flatten}(F_{\mathrm{local}})\right)\in R^{B\times 196\times 768}.$$

Here, $Z_{\mathrm{local}}$ denotes the patch-level local features extracted by the CNN local branch.

#### 2.3.4. ViT Global Context Branch

**Figure 6:**
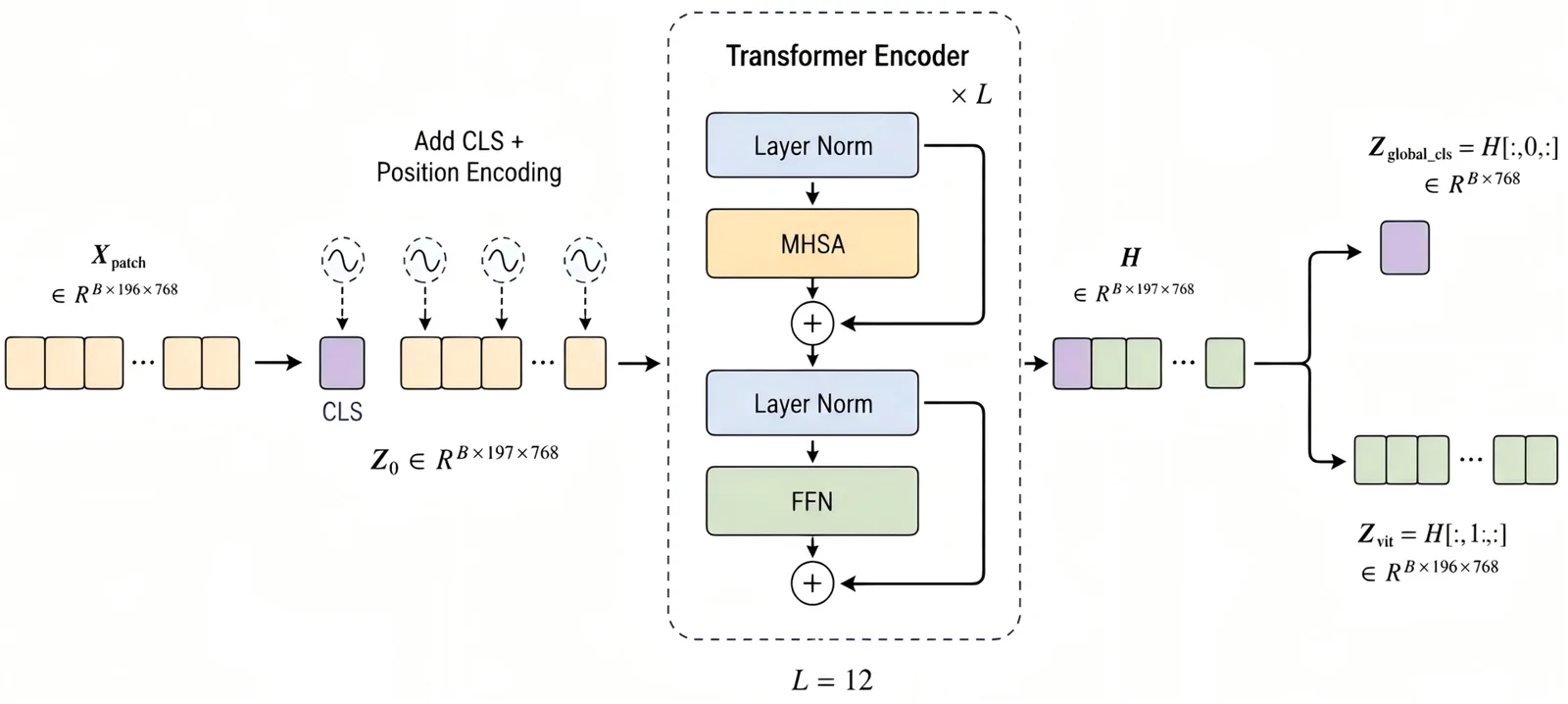
Structure of the vision transformer (ViT) global context branch.

Unlike convolutional neural networks, which mainly focus on local neighborhood structures, ViT can establish relationships between any two patches at the global scale through the self-attention mechanism. This enables the model to better capture the overall spatial distribution patterns of scanpath trajectories, as illustrated in Figure 6.

To obtain a global semantic representation of the entire image, a learnable classification token $x_{\mathrm{cls}}$ is prepended to the patch token sequence. A learnable positional embedding $E_{\mathrm{pos}}$ is then added to the entire token sequence to preserve the spatial position information of patches. Therefore, the initial input representation of the ViT branch is formulated as:

(7) $$Z_{0}=\left[x_{\mathrm{cls}};X_{\mathrm{patch}}\right]+E_{\mathrm{pos}},\;Z_{0}\in R^{B\times (N+1)\times d}=R^{B\times 197\times 768}$$

where [;] denotes concatenation along the sequence dimension, and $E_{\mathrm{pos}}\in R^{B\times (N+1)\times d}$ denotes the positional embedding. The input sequence $Z_{0}$ is then fed into the ViT backbone, which consists of *L* Transformer encoder layers. Each Transformer encoder layer is composed of a multi-head self-attention module (MHSA), a feed-forward network (FFN), layer normalization (LN), and residual connections. For the *l*-th Transformer encoder layer, the computation is defined as:

(8) $$\begin{array}{cc} Z_{l}^{\prime } & =Z_{l-1}+\mathrm{MHSA}\left(\mathrm{LN}\left(Z_{l-1}\right)\right),\\ Z_{l} & =Z_{l}^{\prime }+\mathrm{FFN}\left(\mathrm{LN}\left(Z_{l}^{\prime }\right)\right) \end{array}$$

where l=1,2,…,L. $Z_{l}^{\prime }$ denotes the intermediate representation after the multi-head self-attention module in the *l*-th layer, and $Z_{l}$ denotes the output of the *l*-th Transformer encoder layer. *L* denotes the number of Transformer encoder layers, LN(·) denotes layer normalization, MHSA(·) denotes multi-head self-attention, and FFN(·) denotes the feed-forward network.

In the experiments, the ViT branch adopts the ViT-Base architecture, with L=12 encoder layers. After passing through all Transformer encoder layers, the encoded output sequence is obtained as:

(9) $$H=Z_{L}\in R^{B\times 197\times 768}.$$

Since the first token of the output sequence corresponds to the classification token and the remaining tokens correspond to patch features, *H* is split into two parts:

(10) $$\begin{array}{c} Z_{\mathrm{global}\_\mathrm{cls}}=H\left[:,0,:\right]\in R^{B\times 768},\\ Z_{\mathrm{vit}}=H\left[:,1:,:\right]\in R^{B\times 196\times 768} \end{array}$$

where $Z_{\mathrm{global}\_\mathrm{cls}}$ denotes the global classification feature extracted by the ViT branch, which provides whole-image semantic information for the subsequent classification stage. $Z_{\mathrm{vit}}$ denotes the patch-level features after global contextual modeling. These features capture long-range dependencies among different patches and serve as one input to the subsequent gated fusion module.

#### 2.3.5. Gated Adaptive Fusion Module

**Figure 7:**
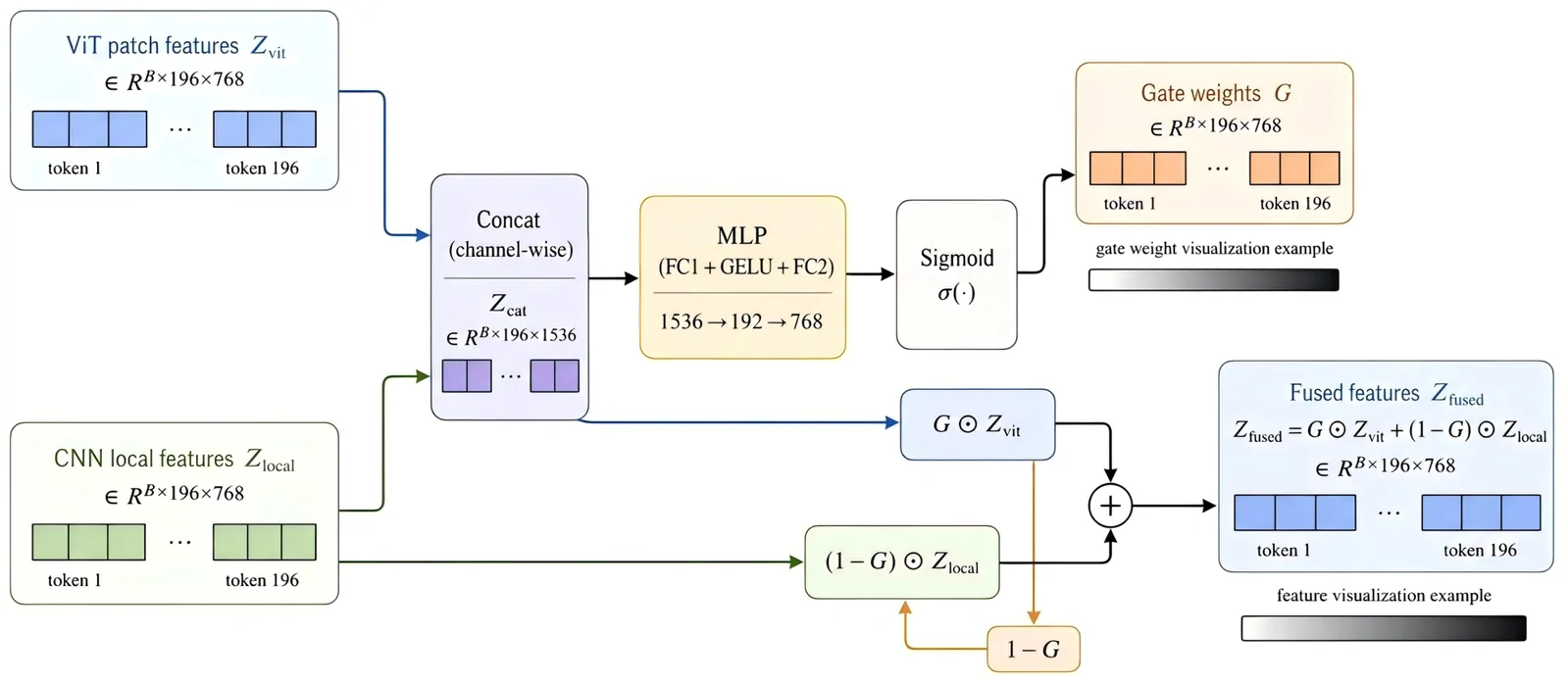
Illustration of the gated adaptive fusion module.

After obtaining the ViT global contextual features and CNN local structural features, this study further designs a gated adaptive fusion module to achieve fine-grained integration of the two feature types at the patch level. Figure 7 shows the two patch-level inputs: $Z_{\mathrm{vit}}$ from the ViT branch and $Z_{\mathrm{local}}$ from the CNN branch. Because they have the same number of tokens and the same feature dimension, token-wise and channel-wise adaptive fusion can be performed.

First, the two features are concatenated along the feature dimension:

(11) $$Z_{\mathrm{cat}}=\left[Z_{\mathrm{vit}};Z_{\mathrm{local}}\right]\in R^{B\times 196\times 1536}$$

The concatenated feature is then fed into a gating network composed of two fully connected layers, and a Sigmoid function is used to generate the gating weights:

(12) $$G=\sigma \left(\mathrm{MLP}\left(Z_{\mathrm{cat}}\right)\right),\;G\in R^{B\times 196\times 768}$$

Here, σ(·) denotes the Sigmoid activation function, and MLP(·) denotes a multilayer perceptron consisting of two fully connected layers. The detailed process can be written as:

(13) $$\begin{array}{cc} H & =\delta \left(Z_{\mathrm{cat}}W_{1}+b_{1}\right),\;H\in R^{B\times 196\times 192},\\ U & =HW_{2}+b_{2},\;U\in R^{B\times 196\times 768},\\ G & =\sigma \left(U\right),\;G\in R^{B\times 196\times 768} \end{array}$$

Each element in *G* ranges from 0 to 1 and controls the relative contribution of the ViT global contextual feature and CNN local structural feature at different tokens and feature channels.

When a gating weight is closer to 1, the model tends to retain more global contextual information extracted by the ViT branch. When a gating weight is closer to 0, the model tends to retain more local structural information extracted by the CNN branch. The final gated patch-level feature is obtained as:

(14) $$Z_{\mathrm{fused}}=G⊙Z_{\mathrm{vit}}+\left(1-G\right)⊙Z_{\mathrm{local}},\;Z_{\mathrm{fused}}\in R^{B\times 196\times 768}$$

where ⊙ denotes element-wise multiplication, and 1−G denotes the local feature weight complementary to the gating weight.

#### 2.3.6. Classification Module and Loss Function

After global–local feature fusion is completed, the model enters the classification stage.

**Figure 8:**
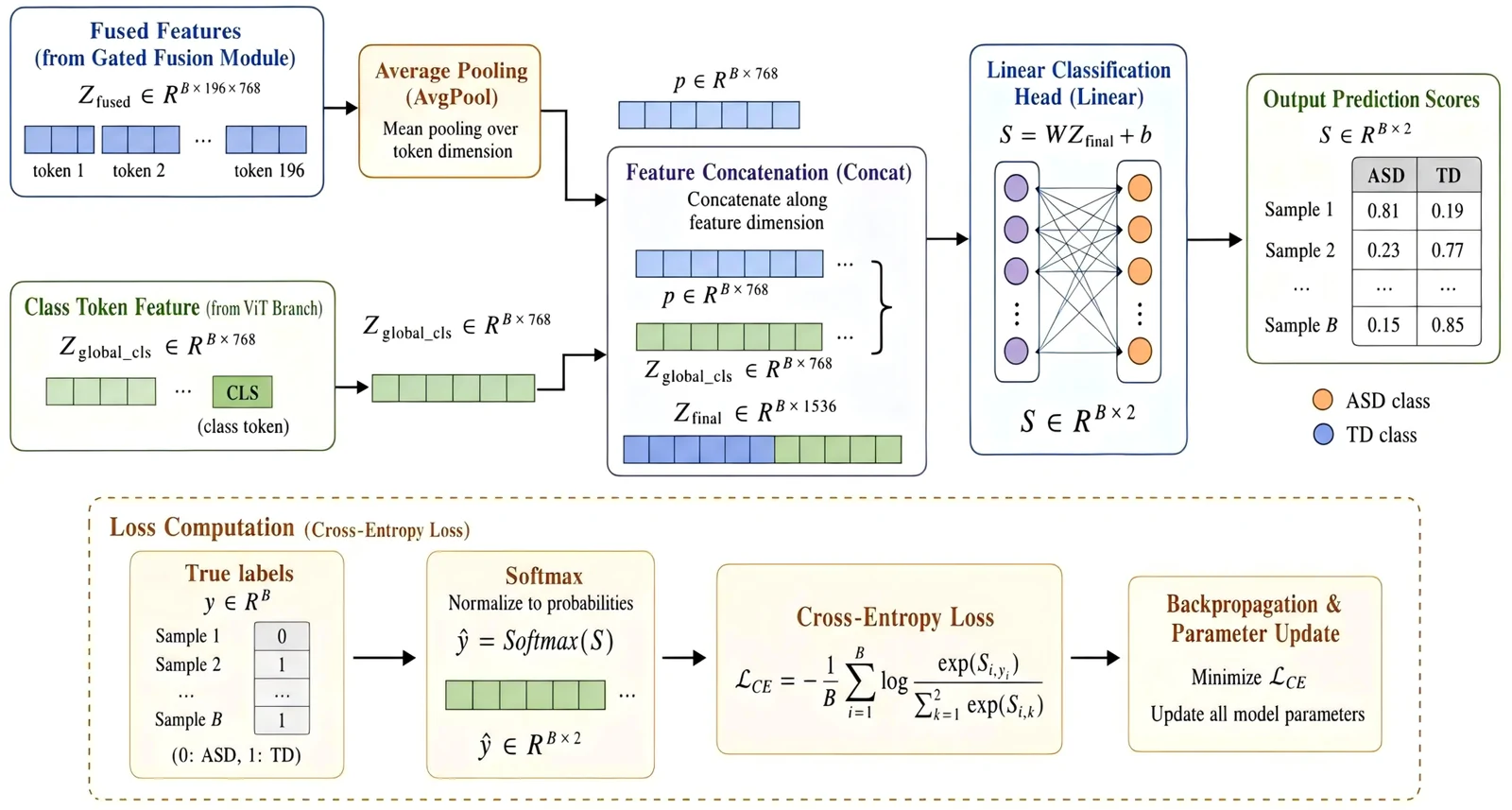
Illustration of the classification module and loss function.Blue and green bars represent two feature branches. Purple circles are hidden-layer neurons, and the vertical orange circles are class-related output nodes. Class information is clearly defined in the prediction-score table and legend. The beige dashed box separates the loss-computation module. The original figure is retained.

Figure 8 shows that $Z_{\mathrm{fused}}$ is first average-pooled along the token dimension to obtain a global fused representation:

(15) $$p=\mathrm{AvgPool}\left(Z_{\mathrm{fused}}\right),\;p\in R^{B\times 768}$$

Meanwhile, the classification-token feature Z_global_cls output by the ViT branch represents whole-image global semantic information. This feature is extracted from the CLS token in the Transformer encoder output sequence and has the following dimension:

(16) $$Z\_\mathrm{global}\_\mathrm{cls}\in R^{B\times 768}$$

To preserve both the gated local–global patch aggregation information and the global semantic information extracted by the ViT branch, this study concatenates the average-pooled fused feature *p* with the classification-token feature $Z_{\mathrm{global}\_\mathrm{cls}}$, producing the final discriminative feature:

(17) $$Z\_\mathrm{final}=\left[p;Z\_\mathrm{global}\_\mathrm{cls}\right],\;Z\_\mathrm{final}\in R^{B\times 1536}$$

The final feature $Z_{\mathrm{final}}$ is then fed into a linear classification head to obtain the unnormalized prediction scores for the two classes, ASD and TD:

(18) $$S=WZ\_\mathrm{final}+b,\;S\in R^{B\times 2},$$

where *W* and *b* denote the weight matrix and bias term of the classification head, respectively. The two elements in the output vector *S* correspond to the predicted scores for the ASD and TD classes.

During training, this study uses the Cross-Entropy Loss for supervised optimization:

(19) $$-\frac{1}{B}\sum \_{i=1}^{B}\log\frac{\exp(S\_i,y\_i)}{\sum \_{k=1}^{2}\exp\left(S_{i,k}\right)}.$$

Here, *B* denotes the batch size, y_i denotes the ground-truth class label of the *i*-th sample, S_i,y_i denotes the model’s prediction score for the true class, and S_i,k denotes the prediction score for the *k*-th class.

By minimizing the cross-entropy loss, the trainable parameters of the model are optimized in a supervised manner. Because this study adopts a frozen ViT backbone strategy under small-sample conditions, the training process mainly updates the CNN local branch, the gated adaptive fusion module, the pre-logits layer, and the classification head, while the ViT backbone parameters remain frozen to reduce the risk of overfitting and improve training stability.

### 2.4. Experimental Setup

#### 2.4.1. Implementation Details

All code, model training, and inference in this study were executed under the following hardware and software environment. The hardware configuration included a 14th generation Intel Core i7 14650HX processor (Intel, Santa Clara, CA, USA), 16 GB RAM, an NVIDIA GeForce RTX 5060 GPU (NVIDIA, Santa Clara, CA, USA), 8 GB of video memory, and a 1 TB solid state drive as secondary storage. The operating system was Windows 11, 64 bit (Microsoft, Redmond, WA, USA). Regarding the software environment, PyTorch 2.11.0 was used as the deep learning framework, with CUDA 12.8 corresponding to the cu128 build of PyTorch. torchvision 0.26.0+cu128 was used for image preprocessing and data augmentation. Other key dependencies included NumPy 2.4.3 for numerical computation and Pillow 12.1.1 for basic image processing. All experiments were conducted under the same environment.

The model input consisted of preprocessed images with a unified size of 3×224×224, and the output layer dimension was set to 2 according to the number of classes. The Cross-Entropy Loss was used to measure the difference between model predictions and ground-truth labels. Labels were encoded as integers and aligned with the output of the classification head.

The ViT-B/16 backbone of GLCF-Net loaded ImageNet-21k pretrained weights to provide relatively stable global visual representations. The CNN local branch and gated fusion module were randomly initialized to learn local trajectory structures and global–local combination patterns in ETSP images. During training, only the training set was used for parameter updates, the validation set was used for model selection and hyperparameter tuning, and the test set was used only for final evaluation. The batch size was set to 16, the number of training epochs was set to 20, and the initial learning rate was $1\times 10^{-4}$. The detailed training configuration is summarized in Table 1.

#### 2.4.2. Evaluation Metrics

This study uses Accuracy, Precision, Recall, F1-score, and ROC-AUC as the main evaluation metrics, which are summarized in Table 2. Accuracy measures the overall classification correctness of the model. Precision and Recall reflect the accuracy and sensitivity of the model for the predicted positive class, namely ASD. F1-score balances Precision and Recall, while ROC-AUC was used to evaluate the model’s discriminative ability across different classification thresholds. In all binary classification evaluations, ASD was treated as the positive class and TD as the negative class; therefore, ROC-AUC was calculated using the predicted probability of the ASD class. Because the dataset has a certain degree of class imbalance at the image level, this study also reports macro-averaged Precision, Recall, and F1-score to avoid interpretation dominated solely by Accuracy.

#### 2.4.3. Repeated Participant-Level Evaluation

Sensitivity to participant partitioning was assessed using repeated stratified five-fold cross-validation. The 54 participants with released ETSP images were divided into five outer folds, and the procedure was repeated twice using master seed 20260716. This seed was fixed before the repeated runs as a reproducibility identifier and was not selected from model performance. Each test fold contained 10 or 11 participants, including 5 or 6 ASD participants and 5 or 6 TD participants; the corresponding training fold contained 43 or 44 participants. Every image from a participant remained in the same fold, and GLCF-Net and all four baselines used the same saved participant assignments. For zero-indexed repeat *r* and fold *f*, the training seed was 20260716+100r+f. Python 3.9, NumPy 2.4.3, and PyTorch 2.11.0 random generators were seeded; cuDNN benchmarking was disabled and deterministic execution was enabled; and each data-loader worker was seeded from the PyTorch worker seed. Run-to-run stochastic training variability was evaluated separately by fitting GLCF-Net five times on the unchanged participant folds of the first repeat. For zero-indexed initialization run *j* and fold *f*, the training seed was 20260716+100j+f. Participant assignments, preprocessing, sampling rule, architecture, hyperparameters, 20-epoch schedule, final-epoch evaluation, and the fixed threshold of 0.5 were held unchanged. For each initialization run, the participant-level metrics were averaged across the same five folds, and variability was summarized as the standard deviation of the five run-level means.

The architecture and training hyperparameters were fixed before cross-validation, and no outer-test prediction was used for model selection or hyperparameter tuning. Each model was trained for 20 epochs, and the final epoch was evaluated without early stopping or threshold optimization. Inputs were resized deterministically to 224×224 pixels and normalized for the corresponding pretrained backbone, with no geometric, color, or sample-mixing augmentation. The training sampler assigned image *i* from participant p(i) and class y(i) a weight proportional to ${\left[n_{p(i)}N_{y(i)}\right]}^{-1}$, where $n_{p(i)}$ is that participant’s image count and $N_{y(i)}$ is the number of training participants in the class. It then sampled, with replacement, a number of images equal to the training-set image count. This gives each participant equal expected contribution within a class and balances the two classes in expectation. The sampler and data-loader generators used the corresponding fold seed.

For VGG19, ResNet-50, and DenseNet-121, the standard torchvision ImageNet-1k weights were loaded and only the final linear classifier was trained. The ViT-B/16 baseline and GLCF-Net loaded the same ImageNet-21k checkpoint and used the same frozen Transformer encoder. ViT-B/16 trained its pre-logits projection and classifier, whereas GLCF-Net additionally trained the CNN local branch and the gated fusion module. All models used batch size 16, cross-entropy loss, AdamW, an initial learning rate of 0.0001, weight decay of 0.0001, and a 20-epoch cosine schedule. No model received a separate outer-fold tuning budget. The CNN and ViT families do not have perfectly matched pretraining sources; consequently, the matched ViT-B/16 comparison and same-backbone ablations provide the most direct architectural controls. Full model-by-model settings and parameter counts are reported in Supplementary Table S2.

For each repeat, ASD probabilities were first averaged across the held-out images of each participant. The two repeat-specific out-of-fold probabilities were then averaged to obtain one probability for each of the 54 participants, and the fixed threshold of 0.5 was applied to that mean. Participant-level Accuracy, Precision, Sensitivity, Specificity, F1-score, and ROC-AUC were calculated from these 54 independent participant records. Two-sided 95% confidence intervals were obtained from 10,000 stratified participant-bootstrap resamples, with ASD and TD participants resampled separately. The mean and standard deviation across the 10 outer folds were retained as descriptive measures of partition sensitivity rather than as independent-sample inference. For the paired Accuracy comparisons, correctness was defined once for each participant after repeat averaging. The Accuracy difference was the mean of the 54 paired correctness differences and therefore changes in increments of 1/54. Its 95% confidence interval was obtained from 10,000 stratified paired-participant bootstrap resamples using seed 20260716. The complete paired 2×2 correctness counts were retained, and two-sided exact McNemar tests were used for the four baseline comparisons, followed by Holm correction across these four tests. ROC-AUC comparisons in the fold-summary table are descriptive and are not assigned paired significance tests. Image-level values were retained as descriptive results and were not treated as independent observations in the participant-level inference.

The no-gate variant replaced the learned gate with equal local/global averaging, the no-CLS variant classified from the mean fused-patch representation alone, and the rotation variant used the configuration described above. These analyses used the same saved participant folds as the complete model. Because these variants were evaluated in the first five-fold repeat, their matched complete-model comparator was recomputed from the same first repeat rather than from both repeats. Gate tensors were averaged across channels and then summarized within participants and diagnostic groups. Incorrectly classified participants were examined using their distance from the 0.5 threshold and between-repeat variability. Finally, online-capable inference was benchmarked after a complete ETSP image was available; gaze acquisition and scanpath rendering time were not included.

## 3. Results

### 3.1. Experimental Results of GLCF-Net

Under the strict participant-independent split, we ensured that eye-tracking scanpath images from the same participant did not appear simultaneously in the training and test sets. Data augmentation was performed only on the training set, and the test set was used exclusively for final performance evaluation. The resulting metrics and participant-wise probability distributions therefore reflect classification performance on unseen participants.

Table 3 summarizes the fixed-split test results. GLCF-Net achieved an Accuracy of 83.52%, a Macro F1-score of 82.68%, and a ROC-AUC of 90.27%. For the TD class, Precision, Recall, and F1-score were 82.76%, 90.57%, and 86.49%, respectively. For the ASD class, Precision, Recall, and F1-score were 84.85%, 73.68%, and 78.87%, respectively. At the class level, the model achieved a higher Recall for TD than for ASD.

To further estimate the uncertainty of test-set performance under the limited number of participants, we additionally computed 95% confidence intervals using participant-level bootstrap resampling. In each bootstrap iteration, test participants were resampled with replacement, and all ETSP images belonging to the sampled participants were used to recalculate the evaluation metrics. This procedure was repeated 1000 times, and the 2.5th and 97.5th percentiles of the bootstrap distribution were used as the lower and upper bounds of the 95% confidence interval. The resulting 95% confidence intervals for GLCF-Net on the participant-independent test set are summarized in Table 4.

### 3.2. Frame-Level Prediction Probability Distribution by Participant

**Figure 9:**
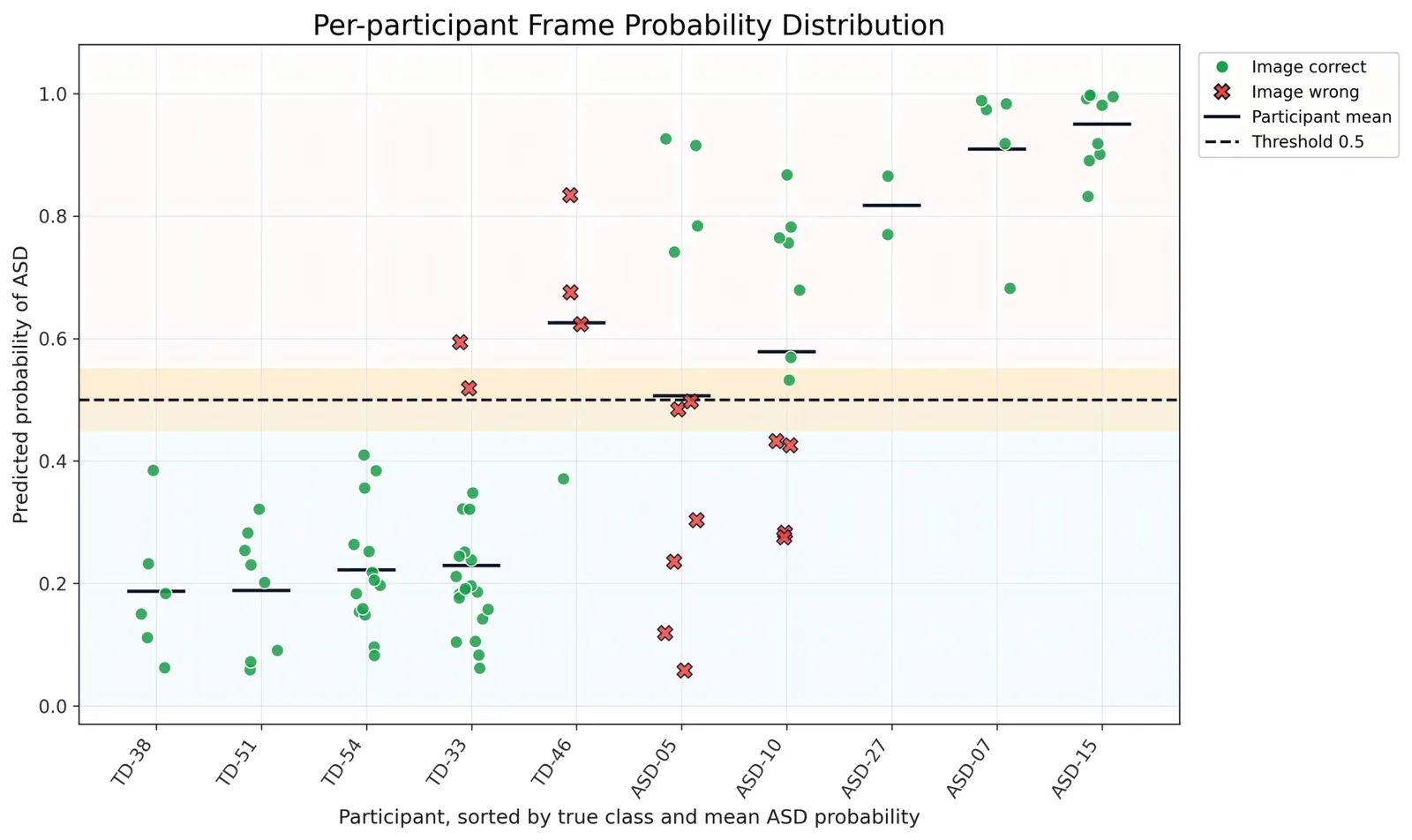
Frame-level prediction probability distribution by participant. The horizontal axis represents different participant IDs in the test set, and the vertical axis represents the model’s predicted probability for the ASD class. Green circles denote correctly predicted image-level samples, red crosses denote incorrectly predicted image-level samples, black short horizontal bars denote the average prediction probability across all images of a given participant, and the dashed line represents the classification threshold of 0.5.

Figure 9 summarizes the frame-level prediction probabilities for each test participant. The probabilities for TD participants are mainly distributed below the 0.5 threshold, whereas those for ASD participants are generally distributed above it. This indicates that the model can distinguish TD and ASD samples relatively well. From the perspective of participant-level average prediction probability, TD-38, TD-51, TD-54, and TD-33 all have average prediction probabilities below 0.5, whereas ASD-27, ASD-07, and ASD-15 all have average prediction probabilities above 0.5, suggesting that the prediction results for these participants are largely consistent with their true labels.

Several participants nevertheless showed boundary or misclassified patterns. For example, the frame-level prediction probabilities of TD-46 are generally above 0.5, and its participant-level average prediction probability is also above the classification threshold, indicating that it was misclassified as ASD. Some frame-level prediction probabilities of ASD-05 and ASD-10 are close to or below 0.5, indicating certain fluctuations across different images within these two participants.

### 3.3. ROC Curve and Confusion Matrix Analysis

**Figure 10:**
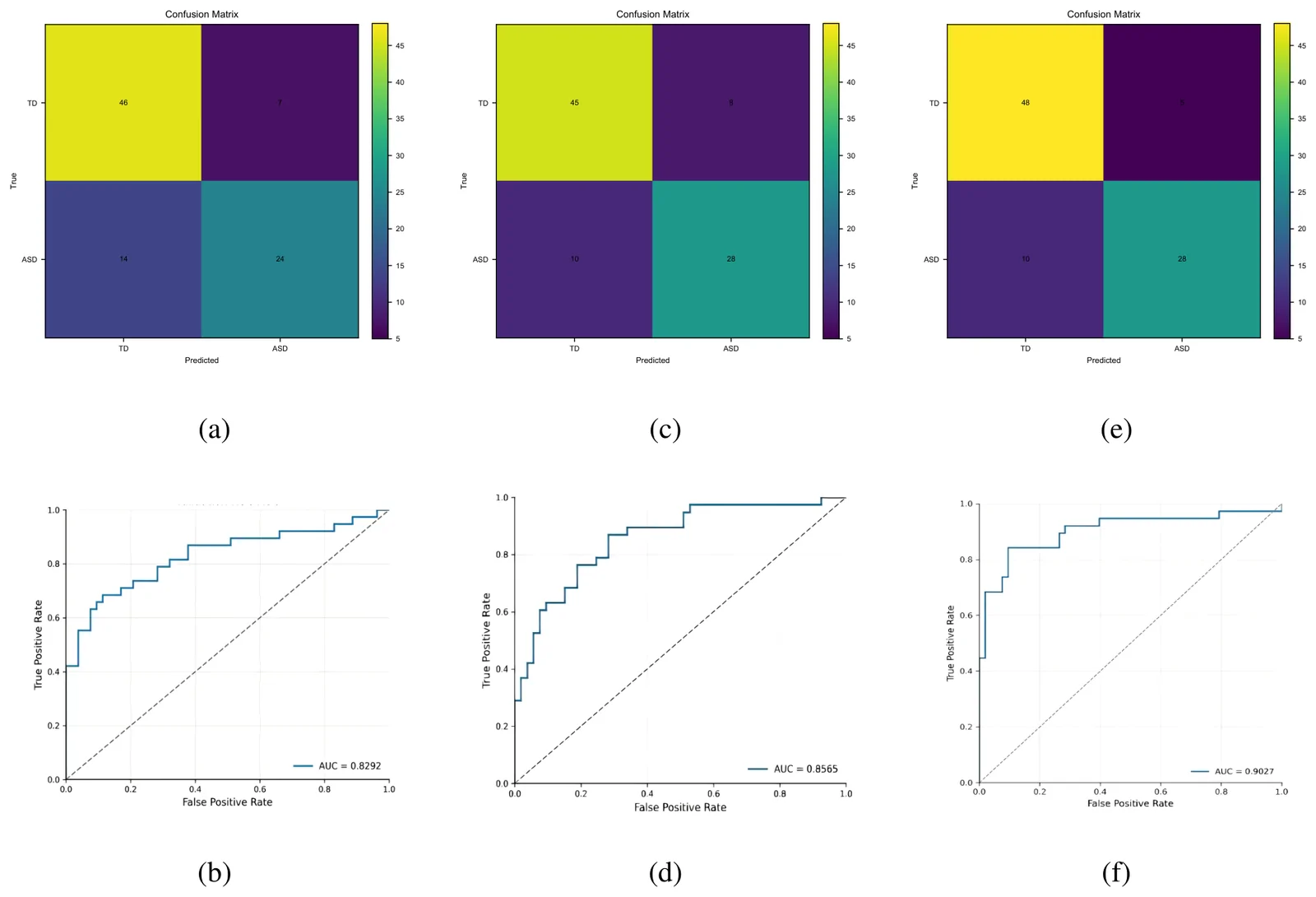
Confusion matrices and ROC curves of different GLCF-Net ablation variants. The top row shows the confusion matrices, and the bottom row shows the ROC curves. (**a**,**b**) ViT-only model with the convolutional branch removed. (**c**,**d**) GLCF-Net without the gated adaptive fusion module. (**e**,**f**) Complete GLCF-Net.

Figure 10e,f presents the confusion matrix and ROC curve for GLCF-Net, respectively. The confusion matrix summarizes the correct and incorrect test-set classifications. Among the 91 test samples, the model correctly classified 76 samples. Specifically, 48 of the 53 TD samples were correctly identified, yielding a TD Recall of 90.57%, while 28 of the 38 ASD samples were correctly identified, yielding an ASD Recall of 73.68%. The ROC curve characterizes discrimination across classification thresholds, with an AUC of 90.27% on the test set.

### 3.4. Performance Comparison with Baseline Models

To validate the performance of GLCF-Net in ETSP scanpath classification, we selected VGG19, ResNet-50, DenseNet, and ViT as comparison models. All models were tested using the same data splitting strategy and evaluation metrics.

Table 5 compares GLCF-Net with the four baseline models on the fixed test set. GLCF-Net achieved the highest values across all five metrics: Accuracy, Precision, Recall, F1-score, and AUC. Specifically, GLCF-Net achieved an Accuracy of 83.52%, exceeding ResNet-50, VGG19, DenseNet, and ViT by 6.15, 9.34, 8.34, and 6.60 percentage points, respectively. Its F1-score reached 82.68%, improving over the above models by 6.80, 9.57, 8.36, and 7.19 percentage points, respectively. Its AUC reached 90.27%, exceeding ResNet-50, VGG19, DenseNet, and ViT by 7.63, 9.85, 7.59, and 7.35 percentage points, respectively.

### 3.5. Ablation Study

We designed three groups of ablation experiments to verify the contribution of each module in GLCF-Net to classification performance. The first group retains only the ViT global branch to examine the performance of single global modeling. The second group removes the gated adaptive fusion module from GLCF-Net and adopts a non-gated fusion strategy. The third group retains the CNN local branch, ViT global branch, and gated adaptive fusion module, forming the complete GLCF-Net. All three groups use the same data split, input size, training strategy, and evaluation metrics.

#### 3.5.1. Effect of the CNN Local Branch

To analyze the role of the CNN local branch, a control model retaining only the ViT global branch, namely the ViT-only model, was constructed. Figure 10a,b presents the confusion matrix and ROC curve for the ViT-only model, respectively. The confusion matrix showed 70 correct classifications among 91 test samples, corresponding to an Accuracy of 76.92% and a Macro F1-score of 75.49%, while the ROC curve yielded an AUC of 82.92%. Specifically, 46 of the 53 TD samples were correctly identified, yielding a TD Recall of 86.79%, whereas 24 of the 38 ASD samples were correctly identified, yielding an ASD Recall of 63.16%. Compared with the complete GLCF-Net, the ViT-only model showed decreases in overall accuracy and ASD recall.

#### 3.5.2. Effect of the Gated Adaptive Fusion Module

To evaluate the effect of the gated adaptive fusion module on the classification performance of GLCF-Net, we constructed a control model by removing the gated adaptive fusion module. This model retains both the CNN local branch and the ViT global branch, but does not use the gating mechanism to adaptively weight and fuse the two feature streams.

Figure 10c,d presents the confusion matrix and ROC curve for the non-gated model, respectively. The confusion matrix showed 73 correct classifications among 91 test samples, corresponding to an Accuracy of 80.22% and a Macro F1-score of 79.51%, while the ROC curve yielded an AUC of 85.65%. Specifically, 45 of the 53 TD samples were correctly identified, yielding a TD Recall of 84.91%, whereas 28 of the 38 ASD samples were correctly identified, yielding an ASD Recall of 73.68%. Compared with the complete GLCF-Net, the non-gated model showed decreases in Accuracy, Macro F1-score, and AUC.

#### 3.5.3. Summary of Ablation Results

Table 6 summarizes the fixed-split ablation results. The complete GLCF-Net achieved the best overall performance in Accuracy, Macro Precision, Macro Recall, Macro F1-score, and ROC-AUC. Specifically, the complete GLCF-Net achieved an Accuracy of 83.52%, exceeding the ViT model and the non-gated GLCF model by 6.60 and 3.30 percentage points, respectively. Its Macro F1-score reached 82.68%, improving over the ViT model and the non-gated GLCF model by 10.58 and 3.17 percentage points, respectively.

At the class level, the complete GLCF-Net achieved higher Precision, Recall, and F1-score for the TD class than the non-gated GLCF model. For the ASD class, the complete GLCF-Net achieved higher Precision and F1-score than both the ViT model and the non-gated GLCF model. Its ASD Recall was 73.68%, the same as that of the non-gated GLCF model and higher than the 63.16% achieved by the ViT model. In addition, the complete GLCF-Net achieved a ROC-AUC of 90.27%, outperforming both the ViT model and the non-gated GLCF model. Overall, the complete GLCF-Net achieved the best results on most evaluation metrics.

### 3.6. Repeated Participant-Level Performance

Repeated participant assignment was used to evaluate performance stability after aggregating predictions at the child level. The grouping audit found no participant overlap between any training and outer-test fold, and each participant received two out-of-fold predictions.

**Figure 11:**
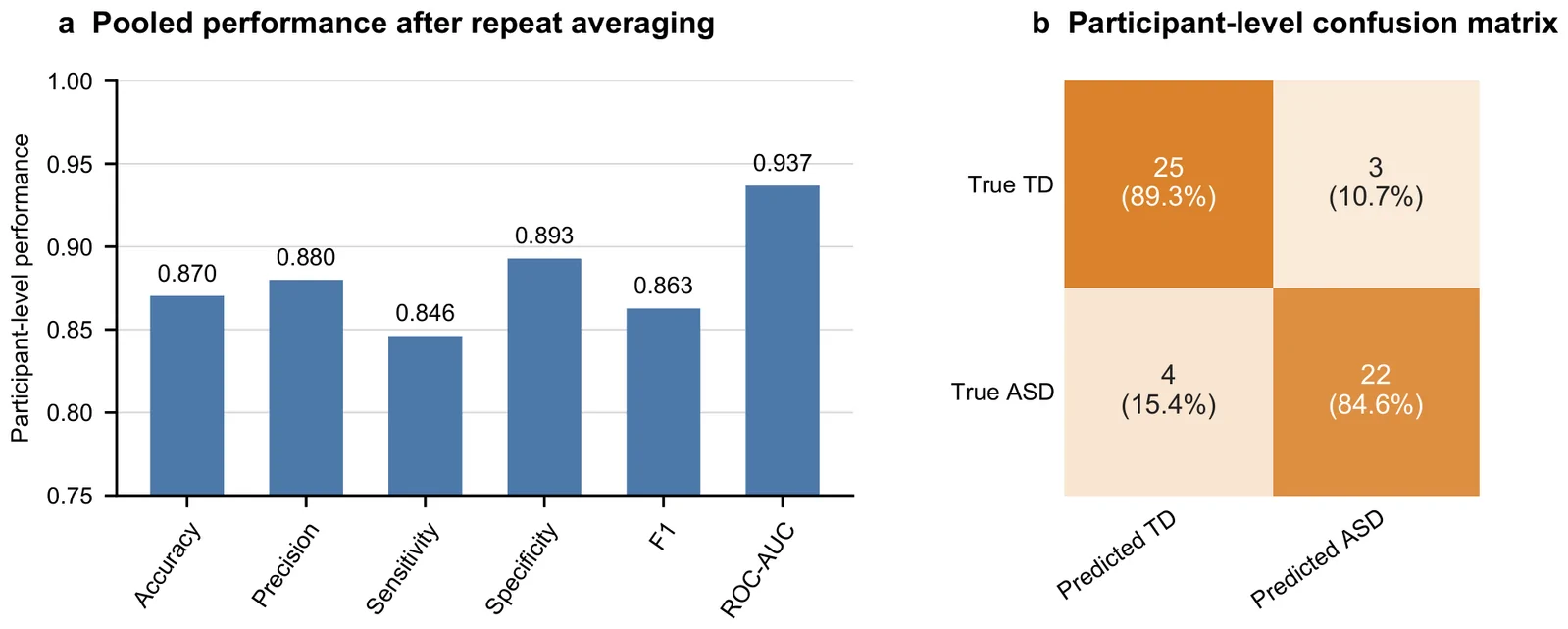
GLCF-Net performance across two repetitions of five-fold participant-level evaluation. (**a**) Pooled Accuracy, Precision, Sensitivity, Specificity, F1-score, and ROC-AUC after averaging each participant’s two repeat-specific out-of-fold probabilities. (**b**) Pooled participant-level confusion matrix obtained by applying the fixed threshold of 0.5 to those probabilities. Counts are accompanied by row percentages.

Across the 10 outer folds, GLCF-Net achieved mean Accuracy 0.870, F1-score 0.859, and ROC-AUC 0.936, with standard deviations of 0.049, 0.045, and 0.028, respectively. After the two out-of-fold probabilities were averaged for each participant, the fixed threshold yielded 25 correctly classified and 3 misclassified TD participants, together with 22 correctly classified and 4 misclassified ASD participants. The corresponding pooled estimates were Accuracy 0.870, Precision 0.880, Sensitivity 0.846, Specificity 0.893, F1-score 0.863, and ROC-AUC 0.937. Participant-bootstrap 95% confidence intervals were 0.778–0.944 for Accuracy, 0.756–0.945 for F1-score, and 0.866–0.995 for ROC-AUC. Figure 11 presents the pooled endpoints and integer confusion matrix, while the 10 fold-specific results are reported in Supplementary Table S3.

GLCF-Net produced the highest fold-averaged Accuracy, F1-score, and ROC-AUC under the reported configurations. Its Accuracy, F1-score, and ROC-AUC had standard deviations of 0.049, 0.045, and 0.028 across the 10 folds, respectively. Participant assignments are reported in Supplementary Table S1, and the fold-specific results are reported in Supplementary Table S3.

### 3.7. Repeated-Fold Model Comparisons

**Figure 12:**
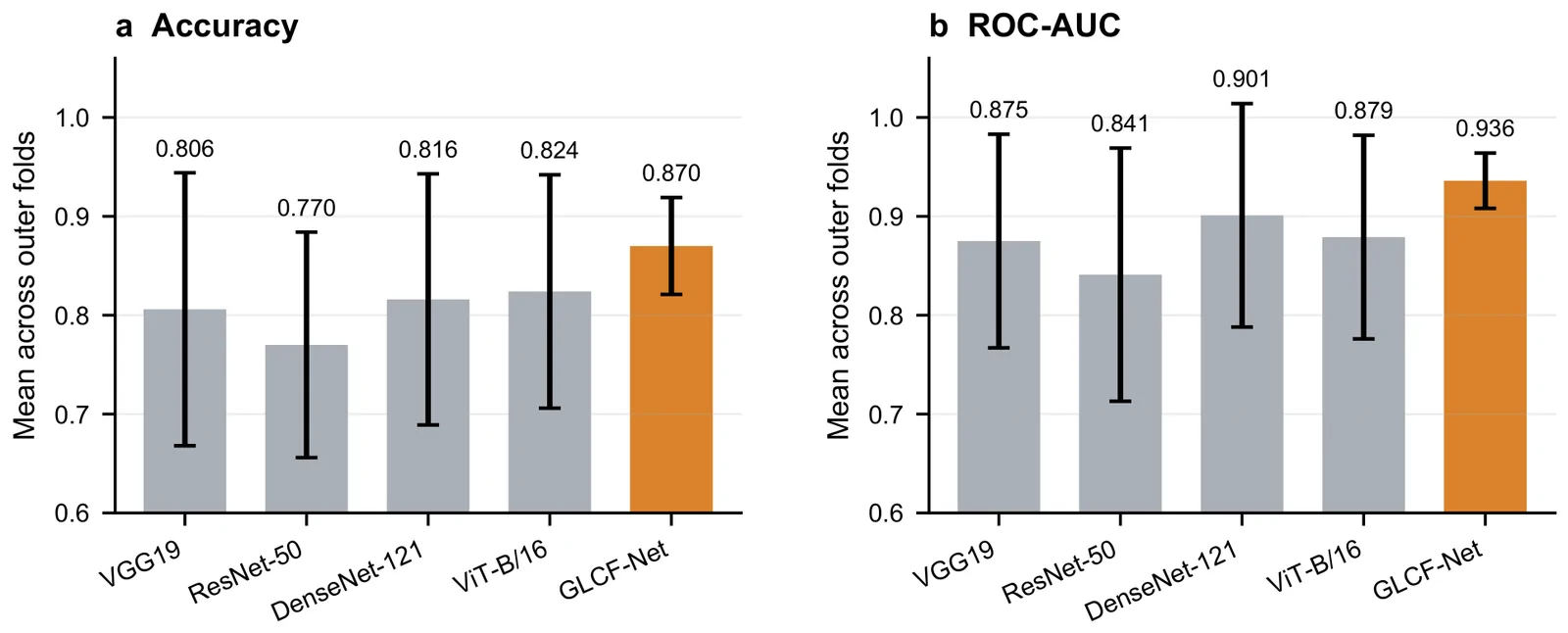
Participant-level Accuracy and ROC-AUC across 10 outer folds. Subplot (**a**) shows Accuracy results, and subplot (**b**) shows ROC-AUC results. Bars represent fold-wise means, and error bars denote standard deviations across folds.

Figure 12 compares models across the repeated outer folds. Under the reported configurations, GLCF-Net exceeded the strongest baseline mean by 0.046 for Accuracy and 0.035 for ROC-AUC. Its observed standard deviations were smaller, but these fold summaries are not independent replication studies and are interpreted only as descriptive partition sensitivity. At the participant level after repeat averaging, the Accuracy differences relative to VGG19, ResNet-50, DenseNet-121, and ViT-B/16 were 4/54, 6/54, 5/54, and 2/54, respectively. Each paired confidence interval included zero, and none of the four exact tests was significant after Holm correction; the fold-averaged ROC-AUC values in Table 7 remain descriptive. Detailed pairwise accuracy comparisons after repeat averaging are summarized in Table 8.

### 3.8. Model Components, Classification Errors, and Inference Time

Table 9 reports participant-level ablations and rotation sensitivity using the first saved five-fold repeat for every row. Recomputing the complete-model summary on these same folds yielded Accuracy 0.869, F1-score 0.864, and ROC-AUC 0.932. Equal fusion without the learned gate yielded 0.842, 0.834, and 0.910; removing the CLS vector yielded 0.831, 0.818, and 0.902; and rotation yielded 0.851, 0.843, and 0.919, respectively. On the unchanged participant folds, the five complete-model initialization runs yielded fold-averaged Accuracy values of 0.869, 0.869, 0.869, 0.851, and 0.889; the corresponding F1-scores were 0.864, 0.851, 0.872, 0.836, and 0.893, and the ROC-AUC values were 0.932, 0.931, 0.923, 0.924, and 0.925. The across-run means and standard deviations were 0.869±0.014, 0.863±0.021, and 0.927±0.004, respectively; the run-level results and seeds are reported in Supplementary Table S8. This initialization analysis was descriptive, and no inferential hypothesis test was applied to the five run-level summaries. The ablation differences were also reported descriptively because each variant was fitted once per fold and the five fold-level estimates shared overlapping training sets rather than representing independent experimental replicates.

**Figure 13:**
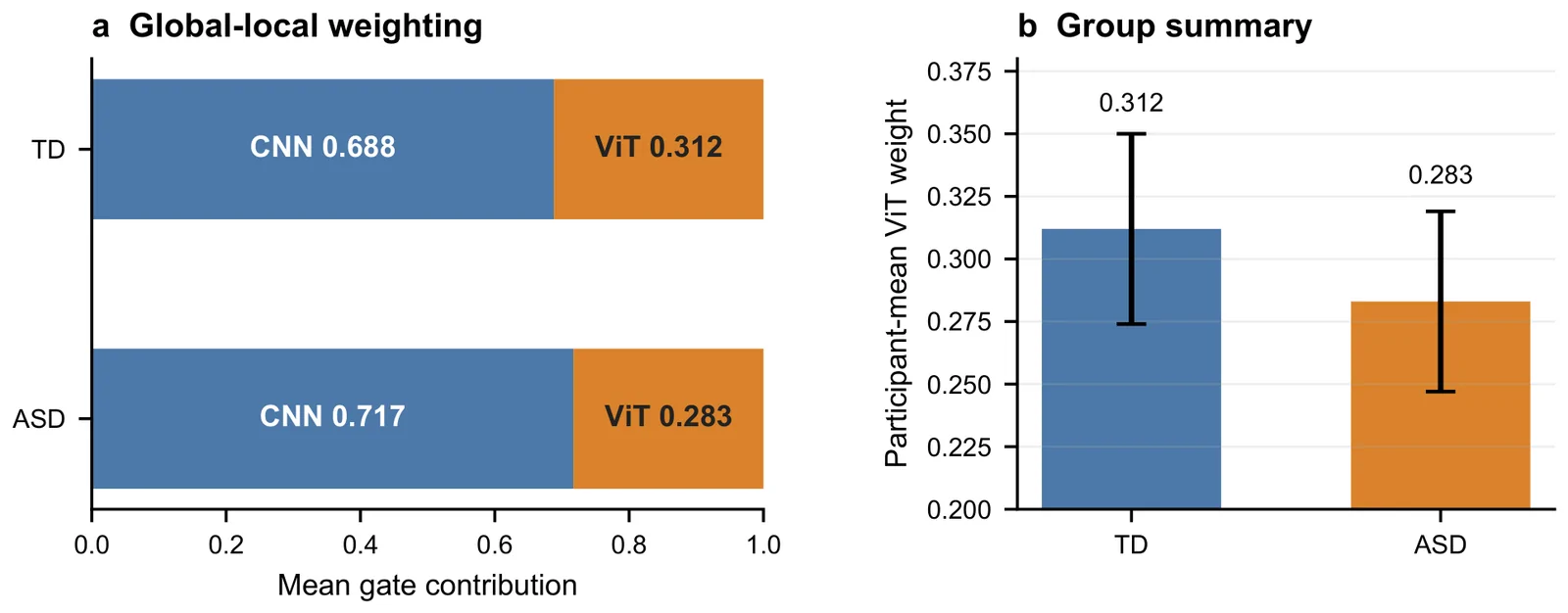
Participant-level GLCF-Net gate analysis. (**a**) Mean CNN and ViT branch weights, which sum to one. (**b**) Participant-mean ViT weights for the TD and ASD groups; error bars show standard deviations. These values describe computational feature mixing and are not biological salience measures.

The learned gate is defined so that one weights the ViT token and zero weights the CNN token. Participant-mean ViT weights were 0.312 (SD 0.038) for TD and 0.283 (SD 0.036) for ASD (Figure 13). The exploratory Mann–Whitney statistic was 244.0 with an uncorrected p value of 0.038. Both means were below 0.5, indicating greater average weighting of the local branch. This analysis summarizes participant- and group-level gate values only; patch-wise spatial gate maps were not evaluated. The group comparison therefore does not identify biological regions or exclude dataset-specific artifacts.

Seven of the 54 participants were misclassified after repeat averaging: ASD-05, ASD-16, ASD-10, and ASD-22, together with TD-45, TD-44, and TD-49. Three mean probabilities were within 0.05 of the fixed threshold, and ASD-22 had a between-repeat probability standard deviation above 0.20. Their repeat-specific probabilities and image counts are reported in Supplementary Table S5.

The online-capable implementation processed a completed ETSP image on the RTX 4090 in a mean model-only time of 6.782 ms (95th percentile, 6.832 ms). Mean preprocessing-plus-inference time was 10.905 ms (95th percentile, 12.097 ms), and batch-16 throughput was 780.36 images per second. These timings start only after a complete ETSP image has been produced and do not include gaze acquisition or scanpath rendering.

## 4. Discussion

This study focuses on ASD auxiliary screening in children and proposes a global–local collaborative fusion network, named GLCF-Net, based on eye-tracking scanpath images. The model was evaluated under a strict participant-independent split to examine its ability to distinguish visual attention patterns between children with ASD and typically developing (TD) children. Unlike conventional random image-level splitting, participant-independent splitting ensures that all scanpath images from the same child appear in only one of the training, validation, or test sets. This setting reduces the risk that the model memorizes participant-specific eye-movement patterns and provides a more cautious evaluation of its performance on unseen participants. Therefore, results obtained under this setting are more informative for assessing cross-participant auxiliary screening performance.

The repeated participant-level evaluation characterized sensitivity to participant partitioning. GLCF-Net achieved mean Accuracy 0.870 and ROC-AUC 0.936, with standard deviations of 0.049 and 0.028 across the outer folds. The corrected paired analysis did not show an Accuracy difference from any individual baseline after Holm correction; the observed fold-averaged performance differences are therefore interpreted descriptively. The separate fixed-fold analysis produced across-initialization standard deviations of 0.014 for Accuracy, 0.021 for F1-score, and 0.004 for ROC-AUC. These values quantify stochastic training variability conditional on one saved set of participant folds and do not establish stability across independent cohorts.

### 4.1. Classification Performance of GLCF-Net Under Participant-Independent Splitting

Under the strict participant-independent setting, GLCF-Net achieved an Accuracy of 83.52%, a Macro F1-score of 82.68%, and a ROC-AUC of 90.27% on the test set. Its overall performance was higher than that of the selected CNN-based baselines and the single-branch ViT model. Compared with random image-level splitting, participant-independent splitting avoids assigning different scanpath images from the same child to both training and test sets, thereby reducing the risk of performance overestimation caused by participant-level information overlap.

Previous ETSP-based studies have reported high classification performance under random splitting or cross-validation after data augmentation. However, such results may be affected by sample-level leakage or participant-level overlap. In contrast, Cilia et al. [10] reported an accuracy of approximately 71% under strict participant-independent splitting, while Kanhirakadavath et al. [11] reported an overall Accuracy of 72.88% on a mini dataset containing one trial per participant. In a similar evaluation context that emphasizes participant independence, the proposed model still achieved relatively good performance, suggesting that jointly modeling global fixation distribution and local trajectory structure is useful for ETSP-based ASD auxiliary screening.

From the class-wise results, GLCF-Net achieved a recall of 90.57% for the TD class, which was higher than the ASD recall of 73.68%. This indicates that the model identified the visual attention patterns of TD children more consistently, whereas some ASD samples were still missed, with certain ASD scanpath images being incorrectly classified as TD. From an eye-movement perspective, this may suggest that the visual attention patterns of some ASD participants partially overlapped with those of TD participants, particularly in terms of fixation distribution, scanpath density, or local trajectory organization. This observation is consistent with the substantial heterogeneity of ASD-related gaze behaviors [20,21] and further suggests that a single ETSP image may not fully capture participant-level variability in visual attention strategies.

### 4.2. Effect of Global–Local Collaborative Modeling

The baseline results show that ResNet-50 achieved relatively better Accuracy and F1-score among the CNN-based models, suggesting that residual convolutional structures can effectively capture local trajectory morphology, path density, and spatial neighborhood patterns in ETSP images. Meanwhile, the ViT model showed a certain advantage in ROC-AUC, indicating that global self-attention is helpful for modeling long-range dependencies among image patches and the overall fixation distribution [25]. These results suggest that both local trajectory structures and global spatial layouts in ETSP images may contain discriminative information for ASD/TD auxiliary recognition. Based on this observation, GLCF-Net introduces a lightweight residual convolutional branch and combines it with a ViT-based global branch for collaborative feature modeling.

The performance of ResNet-50 indicates the potential usefulness of residual convolutional structures for ETSP local feature extraction, but does not isolate the contribution of the lightweight residual block in GLCF-Net. The ablation experiments therefore evaluated the role of the local branch. When only the ViT global branch was retained, Accuracy, Macro F1-score, and ASD recall were lower than those of the complete GLCF-Net. The lower ASD recall suggests that a single ViT branch may not sufficiently capture local scanpath morphology and fine-grained spatial structures. Because the CNN baselines trained only their final linear classifiers, whereas ViT-B/16 and GLCF-Net trained larger task-specific components, the CNN comparisons do not isolate architecture from trainable capacity. The matched ViT-B/16 comparison and the same-backbone ablations provide the more direct evidence for the added local branch, gate, and CLS readout under the present training protocol.

This finding is consistent with the characteristics of ETSP images. ETSP scanpath images contain not only global fixation distribution and cross-region transition patterns, but also local details such as trajectory density, local path morphology, short-range spatial neighborhood structure, and color variations. A single-scale or single-architecture model may be insufficient to represent these heterogeneous patterns. By using the ViT branch to model global attention relationships and the CNN branch to complement local trajectory structures, GLCF-Net can extract visual attention features from two different perspectives, thereby improving the representation of ASD–TD differences in scanpath images. Therefore, the performance improvement over single CNN or ViT baselines suggests that autism-related visual attention patterns in ETSP images may involve both fine-grained local trajectory characteristics and broader global attention allocation strategies.

This interpretation is computational rather than mechanistic. Prior eye-tracking evidence indicates that ASD-related gaze differences vary by social versus nonsocial content, stimulus type, age, and study design [17,18], and scanpath differences may include both reduced social preference and increased nonsocial preference [19]. The CNN branch can be sensitive to local density, morphology, and short-range patch neighborhoods, while the ViT branch can represent overall layout and long-range spatial relations. Without stimulus-aligned areas of interest or raw fixation timing, however, neither branch can be assigned to a specific oculomotor or biological mechanism.

### 4.3. Interpretation of the Gated Adaptive Fusion Module

The ablation results show that the ungated model outperformed the ViT-only model in Accuracy, Macro F1-score, and ASD recall. This indicates that the introduction of the CNN local branch can improve ETSP image classification even when a relatively simple fusion strategy is used. On this basis, the complete GLCF-Net further incorporates a gated adaptive fusion module and achieves additional improvements in Accuracy, Macro Precision, Macro Recall, Macro F1-score, and ROC-AUC. These results suggest that the gated mechanism has a positive effect on integrating global and local features.

The main function of the gated adaptive fusion module is to dynamically adjust the contribution of ViT global features and CNN local features according to the responses of different samples, patches, and channels [28]. For samples that rely more on the overall fixation layout, the model can assign greater importance to the ViT branch. For samples with more informative local trajectory morphology, path density, or spatial neighborhood structures, the model can retain more information from the CNN branch. Therefore, compared with ungated fusion, the gated module provides a more flexible feature integration mechanism and allows the model to adaptively balance local and global information according to the characteristics of different scanpath images.

However, the effect of the gated module should also be interpreted cautiously. The complete GLCF-Net and the ungated model both achieved an ASD recall of 73.68%. This indicates that although the gated module improved the overall performance, ASD precision, and ASD F1-score, it did not further improve ASD recall. In other words, the current gated fusion strategy mainly improved the overall decision boundary and prediction precision, but its effect on reducing missed ASD cases remains limited. Considering that auxiliary screening tasks require high sensitivity to potential ASD cases, future studies may incorporate class-sensitive loss functions, participant-level probability aggregation, raw temporal eye-tracking features, or multimodal behavioral indicators to further improve the sensitivity of ASD recognition.

Across the repeated participant folds, the fitted gate assigned less than half of its average weight to the ViT branch in both groups. Within the matched first repeat, the equal-fusion, no-CLS, and rotation variants each yielded lower Accuracy, F1-score, and ROC-AUC than the complete model on the same five participant folds. The separate five-run initialization analysis was conducted only for the complete GLCF-Net.

### 4.4. Participant-Level Prediction Stability and Misclassification Analysis

In addition to overall classification metrics and ablation results, the frame-level probability distribution at the participant level provides supplementary evidence for evaluating model stability. Since each participant usually corresponds to multiple ETSP images, the prediction of a single image may be influenced by stimulus content, image quality, scanpath sparsity, or short-term attentional state. Therefore, evaluating the model only by image-level Accuracy or AUC is not sufficient. It is also necessary to examine whether the prediction probabilities of multiple images from the same participant are relatively consistent.

At the participant level, most TD participants showed ASD prediction probabilities below the threshold of 0.5, whereas most ASD participants showed probabilities above this threshold. This indicates that the model produced prediction trends that were generally consistent with the true labels for most unseen participants. Thus, GLCF-Net not only achieved good image-level classification performance, but also showed relatively stable prediction tendencies at the participant level.

Nevertheless, some boundary cases and misclassifications were still observed. For example, TD-46 was misclassified as ASD, suggesting that this participant’s scanpath images may share certain local trajectory patterns or global fixation distributions with ASD samples. In addition, some frame-level prediction probabilities of ASD-05 and ASD-10 were close to or below the classification threshold, indicating relatively large prediction fluctuations across different images from the same ASD participant. This phenomenon suggests that a single ETSP image may not be sufficient to represent the overall visual attention characteristics of a child. The model may still face difficulties when dealing with participants whose visual attention patterns are highly heterogeneous or overlap with those of the other class. Future work may adopt participant-level probability averaging, voting-based fusion, or temporal aggregation strategies to reduce the influence of single-frame prediction fluctuations on the final auxiliary screening result.

Seven of the 54 participants were misclassified after repeat averaging, including ASD-05, ASD-16, ASD-10, ASD-22, TD-45, TD-44, and TD-49. Three errors lay within 0.05 of the decision threshold, and ASD-22 had a between-repeat probability standard deviation of 0.2879. The errors were therefore concentrated in a small subset of boundary or partition-sensitive cases, although the available image data do not identify participant-level clinical causes.

### 4.5. Generalization Boundary, Image Representation, and Future Work

Participant-independent evaluation prevents the same child from appearing in training and evaluation subsets, but it demonstrates only within-dataset generalization to unseen participants under the same acquisition conditions. It does not establish external or clinical generalization across independent datasets, centers, eye trackers, age groups, demographic populations, stimulus sets, or experimental paradigms. The effective sample size of 54 image-contributing participants, rather than 547 repeated images, limits precision. The five initialization runs on unchanged participant folds provided a separate estimate of run-to-run stochastic training variability, but this estimate remains conditional on one saved fold set, the present training configuration, and the available computing environment. It does not replace evaluation across independently sampled cohorts, acquisition systems, or broader training configurations.

ETSP images preserve spatial trajectory geometry, local path density, and some color-encoded progression, but rasterization cannot fully preserve ordered fixation coordinates, timestamps, durations, pupil measures, or stimulus-aligned areas of interest. Sequence-specific architectures such as Gazeformer [29] and the Gaze Scanpath Transformer [30] model ordered gaze information more directly. Future studies should compare image, raw-sequence, and joint representations using identical participants and stimuli, followed by preregistered external validation on independent multi-center cohorts and devices.

The measured inference latency indicates that the implementation can rapidly classify a completed ETSP image. It does not yet constitute closed-loop online eye tracking because gaze acquisition and scanpath-image construction occur before model inference. A future online system should accept streaming gaze coordinates, define a prespecified decision window, and validate end-to-end latency and calibration prospectively.

## 5. Conclusions

This study addressed the task of classifying children with autism spectrum disorder (ASD) and typically developing (TD) children using eye-tracking scanpath (ETSP) images, and proposed a global–local collaborative fusion network, named GLCF-Net. Based on a shared patch embedding layer, the proposed method uses a CNN local branch to extract local scanpath trajectory features and a ViT global branch to model cross-region fixation dependencies. A gated adaptive fusion module is then employed to dynamically integrate these two types of features. GLCF-Net combines local trajectory structures and global fixation distributions and was evaluated for unseen participants from the same dataset under participant-independent splitting.

The experiments were conducted on a public ETSP scanpath image dataset. A strict participant-independent splitting strategy was adopted to prevent scanpath images from the same participant from appearing simultaneously in the training and test sets. The experimental results show that GLCF-Net achieved an Accuracy of 83.52%, a Macro F1-score of 82.68%, and a ROC-AUC of 90.27%. Its overall performance was better than that of baseline models, including VGG19, ResNet-50, DenseNet, and ViT. Compared with existing ETSP-related studies that reported results under participant-independent or more conservative evaluation settings, the proposed method showed relatively good auxiliary recognition performance in terms of Accuracy and ROC-AUC. This suggests that global–local collaborative modeling is effective to some extent under strict cross-participant evaluation.

The ablation experiments further indicate that the CNN local branch can complement the limitation of ViT in modeling local trajectory structures, enabling the model to better capture local spatial details in ETSP images. The gated adaptive fusion module improves the flexibility of integrating global and local features and further enhances the overall classification performance. In addition, the participant-level prediction probability distribution shows that the model can produce prediction trends consistent with the true labels for most unseen participants. However, misclassification still occurs for boundary samples and individuals with highly heterogeneous visual attention patterns.

Overall, GLCF-Net learned ETSP-based visual attention representations under participant-independent splitting, providing auxiliary value for analyzing visual attention patterns in children with ASD. Nevertheless, this study is still limited by the small scale of the public dataset and by the fact that ETSP images compress part of the original temporal eye-tracking information. Future work may incorporate larger-scale and multi-center datasets, as well as raw eye-tracking sequences, AOI-based features, and multimodal behavioral information, to further validate the generalization ability, stability, and interpretability of the model.

After repeat averaging, participant-level Accuracy was 87.0%, F1-score was 86.3%, and ROC-AUC was 93.7%; the corresponding fold standard deviations for Accuracy, F1-score, and ROC-AUC were 0.049, 0.045, and 0.028. Independent, sequence-based, prospective, and clinical validation remains necessary to establish performance across acquisition settings and populations.

## Figures and Tables

**Table 1:** Experimental training configuration.

Parameter	Setting
Input size	224×224
Batch size	16
Number of epochs	20
Optimizer	AdamW
Initial learning rate	$1\times 10^{-4}$
Learning rate scheduler	Cosine annealing
Final learning rate ratio	$1\times 10^{-2}$
Weight decay	$1\times 10^{-4}$
Pretrained backbone	ViT-Base/16 pretrained on ImageNet-21k
Frozen ViT backbone	Yes
Feature concatenation	Yes
Class mapping	0 = TD, 1 = ASD

**Table 2:** Evaluation metrics and their formulas.

Metric	Formula
Accuracy	$\frac{\mathrm{TP}\;+\;\mathrm{TN}}{\mathrm{TP}\;+\;\mathrm{TN}\;+\;\mathrm{FP}\;+\;\mathrm{FN}}$
Precision	$\frac{\mathrm{TP}}{\mathrm{TP}\;+\;\mathrm{FP}}$
Recall	$\frac{\mathrm{TP}}{\mathrm{TP}\;+\;\mathrm{FN}}$
F1-score	$\frac{2\;\times \;\mathrm{Precision}\;\times \;\mathrm{Recall}}{\mathrm{Precision}\;+\;\mathrm{Recall}}$
ROC-AUC	Area under the ROC curve

TP, TN, FP, and FN denote the numbers of true positive, true negative, false positive, and false negative samples, respectively.

**Table 3:** Classification performance of global–local collaborative fusion network (GLCF-Net) on the test set.

Metric	Value (%)
Accuracy	83.52
Macro Precision	83.80
Macro Recall	82.13
Macro F1-score	82.68
TD Precision	82.76
TD Recall	90.57
TD F1-score	86.49
ASD Precision	84.85
ASD Recall	73.68
ASD F1-score	78.87
ROC-AUC	90.27

**Table 4:** Bootstrap confidence intervals of GLCF-Net on the participant-independent test set.

Metric	Value (%)	95% CI (%)
Accuracy	83.52	76.92–89.01
Macro Recall	82.13	73.85–88.74
Macro F1-score	82.68	74.56–89.12

**Table 5:** Classification results of GLCF-Net and comparison models on the eye-tracking scanpath (ETSP) test set.

Model	Accuracy (%)	Macro Precision (%)	Macro Recall (%)	Macro F1 (%)	ROC-AUC (%)
ResNet-50	77.37	76.50	75.50	75.88	82.64
VGG19	74.18	73.90	72.84	73.11	80.42
DenseNet	75.18	74.12	74.66	74.32	82.68
ViT	76.92	77.04	74.98	75.49	82.92
GLCF-Net	83.52	83.80	82.13	82.68	90.27

**Table 6:** Classification results of different model structures on the test set.

Metric	ViT (%)	GLCF Without Gating (%)	GLCF-Net (%)
Accuracy	76.92	80.22	83.52
Macro Precision	77.04	79.80	83.80
Macro Recall	74.97	79.30	82.13
Macro F1-score	75.49	79.51	82.68
TD Precision	76.67	81.82	82.76
TD Recall	86.79	84.91	90.57
TD F1-score	81.42	83.33	86.49
ASD Precision	77.42	77.78	84.85
ASD Recall	63.16	73.68	73.68
ASD F1-score	69.57	75.68	78.87
ROC-AUC	82.92	85.65	90.27

**Table 7:** Mean participant-level performance across two repetitions of five-fold cross-validation. ASD is the positive class.

Model	Accuracy	Precision	Sensitivity	Specificity	F1	ROC-AUC
VGG19	0.806	0.817	0.787	0.830	0.798	0.875
ResNet-50	0.770	0.810	0.693	0.847	0.738	0.841
DenseNet-121	0.816	0.845	0.770	0.867	0.796	0.901
ViT-B/16	0.824	0.847	0.767	0.877	0.801	0.879
GLCF-Net	0.870	0.927	0.810	0.927	0.859	0.936

**Table 8:** Paired participant-level Accuracy comparisons after repeat averaging. GLCF-Net correctly classified 47/54 participants. Each row represents a paired correctness table for the same 54 participants. Counts are reported as $n_{11}/n_{10}/n_{01}/n_{00}$, where $n_{11}$ denotes both models correct, $n_{10}$ GLCF-Net only correct, $n_{01}$ the baseline only correct, and $n_{00}$ both models incorrect. The four counts in each row sum to 54, and the Accuracy difference equals $(n_{10}-n_{01})/54$. Confidence intervals use 10,000 stratified paired-participant bootstrap resamples, and p values are from two-sided exact McNemar tests.

Baseline	Counts	Baseline Acc.	ΔAccuracy	95% CI	Exact p	Holm p
VGG19	41/6/2/5	0.796	0.074	[−0.019, 0.185]	0.289	0.584
ResNet-50	38/9/3/4	0.759	0.111	[0.000, 0.241]	0.146	0.584
DenseNet-121	40/7/2/5	0.778	0.093	[−0.019, 0.204]	0.180	0.584
ViT-B/16	41/6/4/3	0.833	0.037	[−0.074, 0.148]	0.754	0.754

**Table 9:** Matched participant-level ablation and rotation sensitivity results. All rows are fold means from the same first saved five-fold repeat.

Variant	Accuracy	F1	ROC-AUC
GLCF-Net	0.869	0.864	0.932
GLCF-Net, no gate	0.842	0.834	0.910
GLCF-Net, no CLS	0.831	0.818	0.902
GLCF-Net, rotation	0.851	0.843	0.919

## Data Availability

The dataset used in this study is publicly available at Figshare: https://doi.org/10.6084/m9.figshare.20113592.v1. The source code, model configurations, evaluation and online-inference scripts, saved participant-fold assignments, and GLCF-Net participant-level predictions are available at https://github.com/LinLing268/GLCF-Net-JEMR (accessed on 4 August 2026).

## Supplementary Materials

The following supporting information can be downloaded at: https://www.mdpi.com/article/10.3390/jemr19040085/s1. Supplementary Methods and Tables S1–S7: Details of participant-fold assignments, model configurations, fold-level performance metrics, secondary image-level results, participant-level error analysis, gate summaries, and the online-inference benchmark. Supplementary Table S8: Fixed-fold training-initialization analysis, including performance comparisons across different random seeds and weight initialization strategies.

## Abbreviations

The following abbreviations are used in this manuscript:

ASD	Autism Spectrum Disorder
TD	Typically Developing
ETSP	Eye-Tracking Scanpath
CNN	Convolutional Neural Network
ViT	Vision Transformer
GLCF-Net	Global–Local Collaborative Fusion Network
ROC	Receiver Operating Characteristic
AUC	Area Under the Curve
