# Supplementary material for: Participant-Independent Classification of Autism-Related Visual Attention Patterns from Eye-Tracking Scanpath Images Using a Global–Local Fusion Network
Source: J Eye Mov Res. 2026 Aug 10;19(4):85. doi: 10.3390/jemr19040085 (PMC13514376; doi:10.3390/jemr19040085)
Supplement: Supplementary file 1 [file jemr-19-00085-s001.zip › jemr-4445244-supplementary.pdf]

Supplementary Information

Participant-Independent Classification of Autism-Related Visual Attention Patterns from Eye-Tracking Scanpath Images Using a Global–Local Fusion Network

Kun Zhang, JunLing Kong, JunHui Zhang, Shuo Zhang, and Jingying Chen

Supplementary Methods S1: Detailed GLCF-Net specification

For an RGB ETSP image $X \in \mathbb{R}^{B\times 3\times 224\times 224}$, a 16×16 patch embedding produces 196 aligned tokens of width 768:

$$X_{\text{patch}} = \text{PatchEmbed}(X) \in \mathbb{R}^{B\times 196\times 768}. \tag{S1}$$

The same token sequence enters both branches. Sharing the embedding preserves patch identity and channel width, which permits element-wise fusion without interpolation.

Local branch

The token sequence is transposed and reshaped to $F_{\text{patch}} \in \mathbb{R}^{B\times 768\times 14\times 14}$. The local residual block uses two 3×3 convolutions with 768 input and output channels, stride 1, padding 1, batch normalization, and GELU activation:

$$F_{\text{local}} = \text{GELU}\left[F_{\text{patch}} + \text{BN}_2\left(\text{Conv}_2\left(\text{GELU}\left[\text{BN}_1\left(\text{Conv}_1(F_{\text{patch}})\right)\right]\right)\right)\right]. \tag{S2}$$

Flattening and transposition return $Z_{\text{local}} \in \mathbb{R}^{B\times 196\times 768}$.

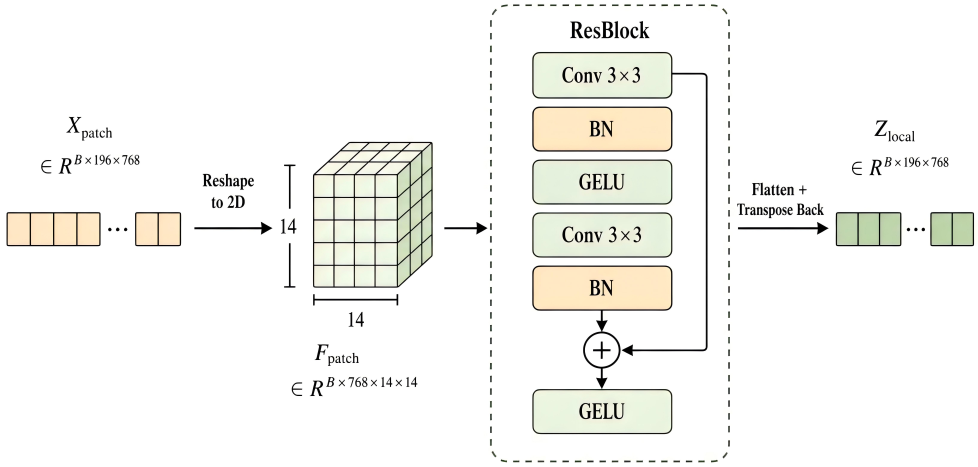

Figure S1: CNN local branch reproduced from the original main-text component diagram for convenient reference.

Global branch

A learnable CLS token is prepended and positional embeddings are added before the 12-layer ViT-B/16 encoder:

$$Z_0 = [x_{\text{cls}}; X_{\text{patch}}] + E_{\text{pos}} \in \mathbb{R}^{B\times 197\times 768}. \tag{S3}$$

The encoder output contains the CLS vector $Z_{cls} \in \mathbb{R}^{B\times768}$. It also contains aligned global patch tokens $Z_{vit} \in \mathbb{R}^{B\times196\times768}$. The patch embedding and Transformer encoder are frozen during formal training.

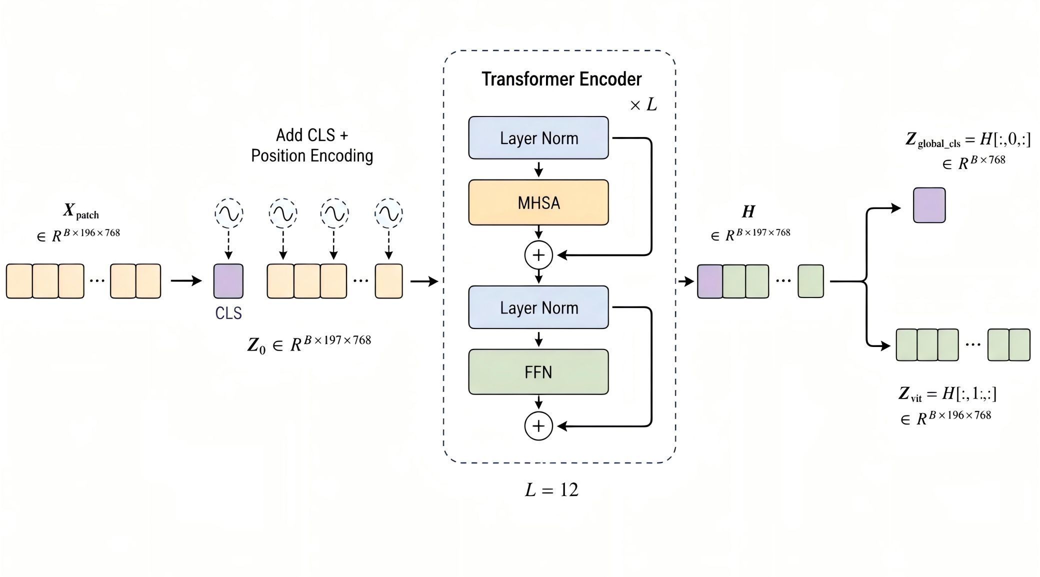

Figure S2: ViT global context branch reproduced from the main text for convenient reference.

Gate and classifier

Concatenated aligned tokens enter a two-layer gate with dimensions 1536 → 192 → 768:

$$G = \sigma\left(\mathrm{MLP}([Z_{vit}; Z_{local}])\right), \tag{S4}$$

$$Z_{fused} = G \odot Z_{vit} + (1 - G) \odot Z_{local}. \tag{S5}$$

The full classifier concatenates mean fused-patch features with the CLS vector:

$$S = W\left[\frac{1}{196}\sum_{j=1}^{196} Z_{fused,j}; Z_{cls}\right] + b, \qquad S \in \mathbb{R}^{B\times2}. \tag{S6}$$

Cross-entropy is minimized over TD (0) and ASD (1). Trainable GLCF-Net components are the residual CNN branch, gate, ViT pre-logits projection, and two-class head. The no-gate ablation fixes $G = 0.5$. The no-CLS ablation removes Z_{cls} and classifies from mean fused-patch features alone.

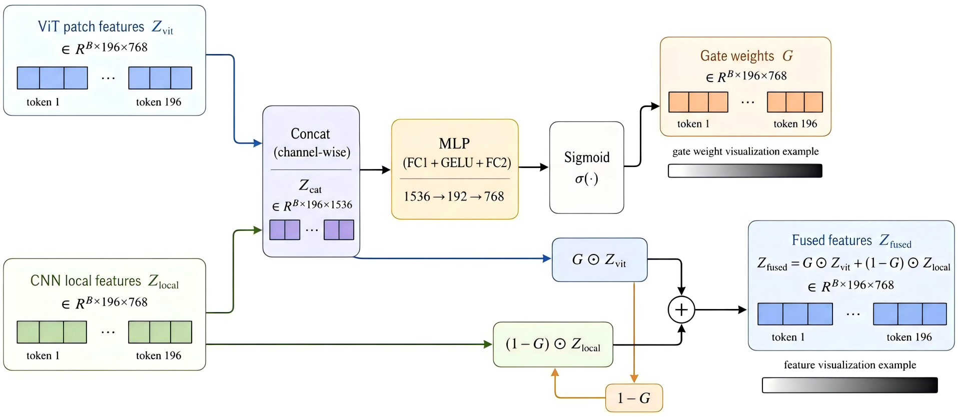

Figure S3: Gated adaptive fusion component reproduced from the main text for convenient reference.

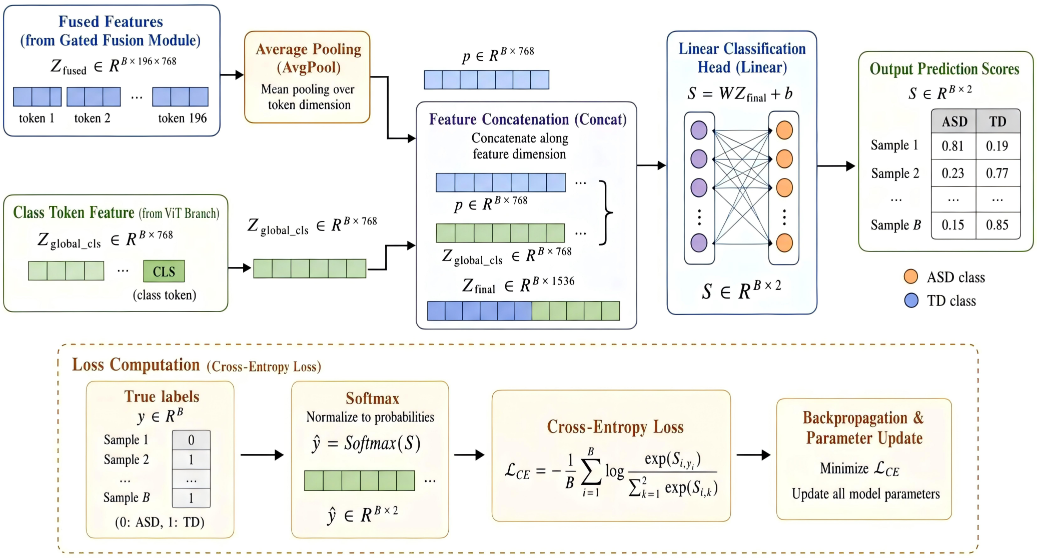

Figure S4: Classification component reproduced from the main text. The implemented readout concatenates the mean fused-patch vector and ViT CLS vector.

Supplementary Methods S2: Evaluation and reproducibility details

The analytic cohort contains 54 participants with released ETSP images. All images from a participant remain in one outer fold. Primary models use two repeats of stratified five-fold participant-level cross-validation and identical saved folds. Ablations use the first five-fold repeat. The master seed is 20260716; it was fixed before the repeated runs and was not selected from model performance. For zero-indexed repeat r and fold f, the training seed is $20260716 + 100r + f$. Python, NumPy, PyTorch, the weighted sampler, and the data-loader generator are seeded for each fold. cuDNN benchmarking is disabled, deterministic execution is enabled, and each data-loader worker is initialized from the PyTorch worker seed. Formal training uses a batch size of 16, cross-entropy, AdamW with an initial learning rate and weight decay of 0.0001, and cosine annealing over 20 epochs. Primary training uses deterministic resizing and normalization only. The rotation sensitivity analysis samples an angle uniformly from -10° to $+10^\circ$ with probability 0.5 within the training fold. Every model is evaluated at its fixed final epoch. No outer-test prediction is used for model, epoch, threshold, or hyperparameter selection. For matched ablation comparisons, the complete-model summary is computed from the same first five-fold repeat used by the no-gate, no-CLS, and rotation variants. Each ablation variant is fitted once per fold. Run-to-run stochastic training variability is assessed separately for the complete GLCF-Net using five initialization runs on the unchanged first-repeat participant folds. For zero-indexed initialization run j and fold f, the training seed is $20260716 + 100j + f$; all other training and evaluation settings remain fixed.

For an image i from participant $p(i)$ and class $y(i)$, the training sampler uses a weight proportional to $[n_{p(i)}N_{y(i)}]^{-1}$, where $n_{p(i)}$ is the participant's image count and $N_{y(i)}$ is the number of training participants in that class. Sampling is performed with replacement, with the number of draws equal to the training-set image count. Thus, participants have equal expected contributions within a class and the classes are balanced in expectation.

Within each repeat, participant ASD probabilities are means of that participant's held-out image probabilities. The two repeat-specific out-of-fold probabilities are then averaged to obtain one record per participant, and the fixed threshold is 0.5. Two-sided 95% confidence intervals use 10,000 stratified participant-bootstrap resamples of these 54 records, with ASD and TD participants resampled separately. The mean and standard deviation across the ten outer folds are retained only as descriptive summaries of partition sensitivity. All models use the same saved participant assignments. For paired Accuracy comparisons, the final binary correctness indicator is defined once per participant after repeat averaging, so Accuracy differences occur in increments of $1/54$. Paired confidence intervals use 10,000 stratified participant-bootstrap resamples with seed 20260716. The full paired correctness table is reported, and two-sided exact McNemar p values are Holm-adjusted across the four baselines. Fold-averaged ROC-AUC comparisons are descriptive.

Supplementary Tables

Table S1: Held-out participant IDs for the two repeated five-fold cycles. ASD is the positive class.

Repeat	Fold	Held-out ASD IDs	Held-out TD IDs
1	1	ASD-04, ASD-07, ASD-11, ASD-14, ASD-23	TD-33, TD-36, TD-40, TD-51, TD-56, TD-58
1	2	ASD-01, ASD-15, ASD-16, ASD-24, ASD-29	TD-37, TD-38, TD-44, TD-49, TD-50, TD-53
1	3	ASD-02, ASD-03, ASD-19, ASD-20, ASD-27	TD-30, TD-35, TD-43, TD-45, TD-54, TD-59
1	4	ASD-06, ASD-08, ASD-09, ASD-10, ASD-12, ASD-17	TD-31, TD-32, TD-34, TD-55, TD-57
1	5	ASD-05, ASD-13, ASD-18, ASD-22, ASD-28	TD-39, TD-46, TD-47, TD-48, TD-52
2	1	ASD-06, ASD-12, ASD-13, ASD-20, ASD-22	TD-34, TD-36, TD-43, TD-51, TD-55, TD-57
2	2	ASD-03, ASD-14, ASD-15, ASD-18, ASD-29	TD-32, TD-35, TD-45, TD-47, TD-49, TD-54
2	3	ASD-01, ASD-07, ASD-11, ASD-19, ASD-23	TD-46, TD-48, TD-50, TD-53, TD-56, TD-58
2	4	ASD-02, ASD-05, ASD-10, ASD-16, ASD-17, ASD-27	TD-30, TD-37, TD-39, TD-40, TD-59
2	5	ASD-04, ASD-08, ASD-09, ASD-24, ASD-28	TD-31, TD-33, TD-38, TD-44, TD-52

Table S2: Model-specific pretraining, fitted components, and parameter counts. All models otherwise use the common protocol in Section 2.4.

Model	Pretraining	Trainable components	Trainable	Total
VGG19	ImageNet-1k (torchvision VGG19 IMAGENET1K_V1)	final linear classification layer	8,194	139,578,434
ResNet-50	ImageNet-1k (torchvision ResNet50 IMAGENET1K_V2)	final linear classification layer	4,098	23,512,130
DenseNet-121	ImageNet-1k (torchvision DenseNet121 IMAGENET1K_V1)	final linear classification layer	2,050	6,955,906
ViT-B/16	ImageNet-21k ViT-B/16 (152 tensors loaded)	pre-logits projection and classification head	592,130	86,390,786
GLCF-Net	ImageNet-21k ViT-B/16 (152 tensors loaded)	pre-logits projection, CNN local branch, gated fusion, CLS+fused-patch classifier	11,656,898	97,455,554
GLCF-Net, no gate	ImageNet-21k ViT-B/16 (152 tensors loaded)	pre-logits projection, CNN local branch, fixed averaging fusion, CLS+fused-patch classifier	11,213,570	97,012,226
GLCF-Net, no CLS	ImageNet-21k ViT-B/16 (152 tensors loaded)	pre-logits projection, CNN local branch, gated fusion, fused-patch classifier	11,655,362	97,454,018
GLCF-Net, rotation	ImageNet-21k ViT-B/16 (152 tensors loaded)	pre-logits projection, CNN local branch, gated fusion, CLS+fused-patch classifier	11,656,898	97,455,554

Table S3: Participant-level metrics across the repeated outer folds. The final GLCF-Net row is the mean ± standard deviation across its ten fold-specific rows. AUC denotes ROC-AUC.

Model	Repeat	Fold	n	Accuracy	F1	AUC
VGG19	1	1	11	1.000	1.000	1.000
VGG19	1	2	11	0.636	0.600	0.667
VGG19	1	3	11	0.727	0.727	0.833
VGG19	1	4	11	0.909	0.909	0.967
VGG19	1	5	10	0.900	0.889	0.800
VGG19	2	1	11	0.818	0.800	0.867
VGG19	2	2	11	0.545	0.545	0.767
VGG19	2	3	11	0.818	0.800	0.967
VGG19	2	4	11	0.909	0.909	0.967
VGG19	2	5	10	0.800	0.800	0.920
ResNet-50	1	1	11	0.909	0.909	1.000
ResNet-50	1	2	11	0.636	0.500	0.600
ResNet-50	1	3	11	0.727	0.667	0.833
ResNet-50	1	4	11	0.818	0.800	0.967
ResNet-50	1	5	10	0.800	0.800	0.840
ResNet-50	2	1	11	0.727	0.667	0.800
ResNet-50	2	2	11	0.545	0.545	0.667
ResNet-50	2	3	11	0.818	0.800	0.933
ResNet-50	2	4	11	0.818	0.800	0.933
ResNet-50	2	5	10	0.900	0.889	0.840
DenseNet-121	1	1	11	0.909	0.909	1.000
DenseNet-121	1	2	11	0.545	0.444	0.667
DenseNet-121	1	3	11	0.818	0.800	0.933
DenseNet-121	1	4	11	0.909	0.909	1.000
DenseNet-121	1	5	10	0.900	0.889	0.800
DenseNet-121	2	1	11	0.636	0.600	0.800
DenseNet-121	2	2	11	0.818	0.833	0.967
DenseNet-121	2	3	11	0.909	0.889	0.967
DenseNet-121	2	4	11	0.818	0.800	1.000
DenseNet-121	2	5	10	0.900	0.889	0.880
ViT-B/16	1	1	11	1.000	1.000	1.000
ViT-B/16	1	2	11	0.636	0.500	0.700
ViT-B/16	1	3	11	0.909	0.889	0.867
ViT-B/16	1	4	11	0.909	0.909	1.000
ViT-B/16	1	5	10	0.800	0.800	0.800
ViT-B/16	2	1	11	0.818	0.800	0.833
ViT-B/16	2	2	11	0.636	0.600	0.767
ViT-B/16	2	3	11	0.818	0.800	0.933
ViT-B/16	2	4	11	0.909	0.909	0.967
ViT-B/16	2	5	10	0.800	0.800	0.920
GLCF-Net	1	1	11	0.909	0.909	0.933
GLCF-Net	1	2	11	0.909	0.889	0.900
GLCF-Net	1	3	11	0.909	0.889	0.933
GLCF-Net	1	4	11	0.818	0.833	0.933
GLCF-Net	1	5	10	0.800	0.800	0.960
GLCF-Net	2	1	11	0.909	0.889	0.933
GLCF-Net	2	2	11	0.818	0.800	0.900
GLCF-Net	2	3	11	0.909	0.889	0.933
GLCF-Net	2	4	11	0.818	0.800	0.933
GLCF-Net	2	5	10	0.900	0.889	1.000
GLCF-Net	1–2	1–5	54	0.870 ± 0.049	0.859 ± 0.045	0.936 ± 0.028

Table S3b: Pooled GLCF-Net performance after averaging each participant's two repeat-specific out-of-fold probabilities. Confidence intervals use 10,000 stratified participant-bootstrap resamples of the 54 participants.

Metric	Estimate	95% CI
Accuracy	0.870	[0.778–0.944]
Precision	0.880	[0.767–1.000]
Sensitivity	0.846	[0.692–0.962]
Specificity	0.893	[0.750–1.000]
F1-score	0.863	[0.756–0.945]
ROC-AUC	0.937	[0.866–0.995]

Table S4: Secondary image-level metrics across the 10 outer folds, shown as mean ± standard deviation. Images are repeated observations and these values were not used for model-comparison inference.

Model	Accuracy	Precision	Sensitivity	Specificity	F1	AUC
VGG19	0.686 ± 0.056	0.603 ± 0.080	0.655 ± 0.063	0.708 ± 0.079	0.626 ± 0.061	0.770 ± 0.049
ResNet-50	0.705 ± 0.039	0.656 ± 0.092	0.585 ± 0.071	0.788 ± 0.079	0.613 ± 0.050	0.759 ± 0.052
DenseNet-121	0.728 ± 0.066	0.668 ± 0.116	0.672 ± 0.096	0.767 ± 0.099	0.664 ± 0.080	0.794 ± 0.062
ViT-B/16	0.731 ± 0.052	0.677 ± 0.101	0.635 ± 0.082	0.796 ± 0.066	0.652 ± 0.076	0.790 ± 0.047
GLCF-Net	0.812 ± 0.032	0.801 ± 0.041	0.782 ± 0.046	0.838 ± 0.039	0.791 ± 0.038	0.892 ± 0.031

Table S5: Participant-level audit of the seven errors obtained after averaging each participant's two repeat-specific out-of-fold ASD probabilities and applying the fixed threshold of 0.5. SD is the sample standard deviation of the two repeat-specific probabilities.

ID	Label	Repeat 1	Repeat 2	Mean	SD	Same decision	Images
ASD-05	ASD	0.4718	0.5057	0.4888	0.0240	No	11
ASD-16	ASD	0.4579	0.4861	0.4720	0.0199	Yes	2
ASD-10	ASD	0.3016	0.3387	0.3202	0.0262	Yes	15
ASD-22	ASD	0.1464	0.5536	0.3500	0.2879	No	5
TD-45	TD	0.5127	0.5482	0.5305	0.0251	Yes	7
TD-44	TD	0.5219	0.5864	0.5542	0.0456	Yes	10
TD-49	TD	0.5276	0.5889	0.5583	0.0433	Yes	8

ASD-05, ASD-16, and TD-45 were within 0.05 of the threshold. ASD-22 had the largest between-repeat variation. The pooled confusion matrix contained 22 correctly classified and 4 misclassified ASD participants and 25 correctly classified and 3 misclassified TD participants.

Table S6: Exploratory participant-level gate summary. Larger values weight the ViT token more strongly.

Group	Mean	SD
ASD	0.283	0.036
TD	0.312	0.038

Exploratory Mann–Whitney $U = 244.0$, uncorrected $p = 0.038$. This is a computational model summary, not evidence of a biological mechanism.

Table S7: Online-capable inference benchmark after a complete ETSP image is available.

Measure	Value
device	cuda:0
gpu	NVIDIA GeForce RTX 4090
input	single completed 224x224 scanpath image
model_only_batch1_ms_mean	6.7824
model_only_batch1_ms_median	6.7695
model_only_batch1_ms_p95	6.8319
end_to_end_batch1_ms_mean	10.9054
end_to_end_batch1_ms_p95	12.0971
batch16_images_per_second	780.3644
scope_note	This benchmark supports rapid inference after a scanpath image has been formed; it does not establish streaming inference from raw gaze coordinates.

Table S8: Fixed-fold training-initialization analysis for the complete GLCF-Net. Every run uses the unchanged participant folds of the first cross-validation repeat. Values are arithmetic means of the same five participant-level outer-fold metrics; SD in the final row is calculated across the five run-level means. The run-level summaries are descriptive, and no inferential hypothesis test was applied.

Initialization run	Fold seeds	Accuracy	F1	ROC-AUC
1	20260716–20260720	0.869	0.864	0.932
2	20260816–20260820	0.869	0.851	0.931
3	20260916–20260920	0.869	0.872	0.923
4	20261016–20261020	0.851	0.836	0.924
5	20261116–20261120	0.889	0.893	0.925
Mean ± SD	—	0.869 ± 0.014	0.863 ± 0.021	0.927 ± 0.004
